# Extracranial ^125^I Seed Implantation Allows Non-invasive Stereotactic Radioablation of Hippocampal Adult Neurogenesis in Guinea Pigs

**DOI:** 10.3389/fnins.2021.756658

**Published:** 2021-11-30

**Authors:** Lily Wan, Rou-Jie Huang, Chen Yang, Jia-Qi Ai, Qian Zhou, Jiao-E Gong, Jian Li, Yun Zhang, Zhao-Hui Luo, Ewen Tu, Aihua Pan, Bo Xiao, Xiao-Xin Yan

**Affiliations:** ^1^Department of Neurology, Xiangya Hospital, Central South University, Changsha, China; ^2^Department of Anatomy and Neurobiology, Central South University Xiangya School of Medicine, Changsha, China; ^3^Medical Doctor Program, Xiangya School of Medicine, Central South University, Changsha, China; ^4^Department of Neurology, Hunan Children’s Hospital, Changsha, China; ^5^Department of Nuclear Medicine, Xiangya Hospital, Central South University, Changsha, China; ^6^Department of Anesthesiology, The 2nd Xiangya Hospital Central South University, Changsha, China; ^7^Department of Neurology, Brain Hospital of Hunan Province, Changsha, China

**Keywords:** adult neurogenesis, brachytherapy, iodine-125 seed, immature neurons, radiation encephalopathy

## Abstract

Adult hippocampal neurogenesis (AHN) is important for multiple cognitive functions. We sort to establish a minimal or non-invasive radiation approach to ablate AHN using guinea pigs as an animal model. ^125^I seeds with different radiation dosages (1.0, 0.8, 0.6, 0.3 mCi) were implanted unilaterally between the scalp and skull above the temporal lobe for 30 and 60 days, with the radiation effect on proliferating cells, immature neurons, and mature neurons in the hippocampal formation determined by assessment of immunolabeled (+) cells for Ki67, doublecortin (DCX), and neuron-specific nuclear antigen (NeuN), as well as Nissl stain cells. Spatially, the ablation effect of radiation occurred across the entire rostrocaudal and largely the dorsoventral dimensions of the hippocampus, evidenced by a loss of DCX^+^ cells in the subgranular zone (SGZ) of dentate gyrus (DG) in the ipsilateral relative to contralateral hemispheres in reference to the ^125^I seed implant. Quantitatively, Ki67^+^ and DCX^+^ cells at the SGZ in the dorsal hippocampus were reduced in all dosage groups at the two surviving time points, more significant in the ipsilateral than contralateral sides, relative to sham controls. NeuN^+^ neurons and Nissl-stained cells were reduced in the granule cell layer of DG and the stratum pyramidale of CA1 in the groups with 0.6-mCi radiation for 60 days and 1.0 mCi for 30 and 60 days. Minimal cranial trauma was observed in the groups with 0.3– 1.0-mCi radiation at 60 days. These results suggest that extracranial radiation with ^125^I seed implantation can be used to deplete HAN in a radioactivity-, duration-, and space-controllable manner, with a “non-invasive” stereotactic ablation achievable by using ^125^I seeds with relatively low radioactivity dosages.

## Introduction

Adult hippocampal neurogenesis (AHN) is of eminent importance to support learning and memory, regulate emotional behavior, and has also been considered as a therapeutic target for neurodegenerative diseases and neuropsychiatric disorders (Aimone et al., 2006; Saxe et al., 2006; Coras et al., 2010; Kang et al., 2016; Anacker and Hen, 2017; Radad et al., 2017; Alam et al., 2018; Micheli et al., 2018; Toda and Gage, 2018; Miller and Sahay, 2019; Toda et al., 2019; Berdugo-Vega et al., 2020; Surget and Belzung, 2021). While AHN exists widely in mammals including human (Altman, 1963; Altman and Das, 1965; McDonald and Wojtowicz, 2005; Amrein et al., 2011; Lieberwirth et al., 2016; Kuhn et al., 2018; Gage, 2019; Wan et al., 2019), its specific roles in various cognitive and behavioral functions remain to be refined. One approach to determine the necessity of AHN in specific neurobiological functions involves behavioral studies in animals with inhibited or depleted formation of the newborn neurons. Three strategies have been used to ablate AHN, including X-ray radiation, genetic ablation, and mitotic inhibition (Shors et al., 2002; Mizumatsu et al., 2003; Saxe et al., 2006; Wojtowicz, 2006; Ben Abdallah et al., 2013; Yeung et al., 2014; Cho et al., 2015; Tang et al., 2017; Bulin et al., 2018; Youssef et al., 2018; Houben et al., 2019; Kostin et al., 2019; Peng et al., 2019; Rivera et al., 2019). X-ray radiation using either large (e.g., clinical radiotherapy) machines or small animal devices is subjected to biohazard concerns and requires cautious preoperative animal care (e.g., anesthesia and management of stress) and detailed design for controlling radiation range and dosage. On the other hand, genetic and pharmacological ablation may be associated with confounding effects derived from cellular changes irrelevant to the blockage of neurogenesis (Saxe et al., 2006; Wojtowicz, 2006).

Based on the principle that radiation can inhibit cell division and damage proliferative cells, ^125^I seeds are used for brachytherapy of multiple solid tumors, such as lung cancer, liver cancer, prostate cancer, kidney cancer, bone metastases, and intracranial tumors (Schwarz et al., 2012; Wang et al., 2016; Zhang et al., 2016; Hirose et al., 2017; Brun et al., 2018; Vigneault et al., 2018; Zhu et al., 2019; Jia et al., 2020; Li et al., 2020; Watson et al., 2020). The seeds are composed of a titanium tube in the center (diameter 0.8 mm, length 4.5 mm, wall thickness 0.05 mm) and a permeable silver rod at the periphery (diameter 0.05 mm, length 3 mm). Clinical studies show that ^125^I seed brachytherapy can be minimally invasive by specific treatment design, with high dose effect targeting the tumor area, but low dose or minimal effect to the surrounding healthy tissue (Schwarz et al., 2012).

In light of the physical properties and clinical applications of ^125^I seeds and reports that X-ray radiation may cause significant and delayed radiation harm to surrounding tissues, multiple low-dose X-ray irradiation is more favorable to the ablation of newborn neurons than a single high-dose treatment (Tada et al., 2000; Xiong et al., 2010). In this study, we attempted to explore the feasibility of extracranial implantation of ^125^I seeds to ablate hippocampal newborn neurons. We previously reported that AHN is increased in female guinea pigs during pregnancy, whereas its functional implication remains to be investigated (Wan et al., 2019). Guinea pigs have a relatively longer lifespan (than mice and rats) and may be suited for future long-term postradiation recovery studies. Therefore, in the current study, we aimed to establish and optimize the dose–effect relationship between extracranial ^125^I seed radiation and AHN, with an effort to determine a potential radiotoxic effect of high-dose radiation on mature neuronal population in the hippocampal formation as well, using female guinea pigs as an experimental model.

## Materials and Methods

### Animals and Experimental Design

Three-month-old female Hartley guinea pigs were obtained from and maintained in the Center for Laboratory Animals of Xiangya School of Medicine during all the experimental procedures before brain perfusion. Animals were housed in a vivarium with controlled temperature, humidity, and lighting (12:12-h light–dark cycle), with free access to standard laboratory chow and water. Fruit or fresh vegetables were also provided daily. The use of animals complied with the National Institutes of Health (NIH) Guide for the Care and Use of Laboratory Animals. All experimental procedures were approved by the Ethics Committee of Xiangya School of Medicine.

Animals were randomly assigned to a sham operation group (*n* = 8) and an experimental group receiving different dosages of ^125^I seeds local radiation. In pilot studies, we implanted unilaterally three 0.8-mCi ^125^I seeds between the scalp and skull above the temporal lobe for 60 days, which were found to have caused too severe neurotoxic injury to mature neurons. In the final experiments, 18 guinea pigs were randomly divided into six groups: A1, B1, C1, A2, B2, and C2 (*n* = 3). We administered 0.3 mCi of extracranial local radiation in group A1, 0.6 mCi in group B1, and 1.0 mCi in group C1 for 30 days. We also administered 0.3 mCi of extracranial local radiation in group A2, 0.6 mCi in group B2, and 1.0 mCi in group C2 for 60 days. The ^125^I tubes were all prescription-ready products used for clinical brachytherapy.

**FIGURE 1:**
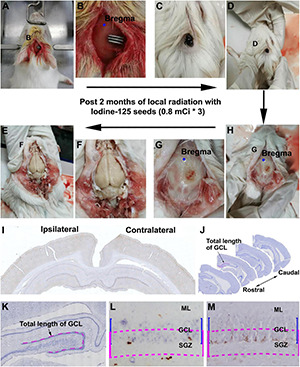
Workflow of experimental procedure and quantification of immunolabeled cells. **(A–E)** Illustrate the experimental procedure from ^125^I seed implantation to brain collection. **(A,B)** Show the initial placement of the ^125^I seeds on the skull (5 mm posterior relative to the bregma and 1 mm lateral relative to the sagittal suture). **(C,D)** Show the presence of ^125^I seeds *in situ* before brain perfusion. **(G,H)** Show the extent of damage to the skull after local radiation. **(E,F)** Show the appearance of the brain after the skull is open. **(I,J)** Show the rostrocaudal range of hippocampus subjected to quantification of immunolabeled positive cells.**(K–M)** Illustrate the method for counting immunolabeled Ki67^+^ **(L)** and DCX^+^ **(M)** cells relative to the length of the granule cell layer (GCL). Cells within the band marked with blue line are counted, which is equal to the depth of the GCL but moved toward the hilus by half of the GCL thickness (purple lines).

We shaved the hair of the female guinea pigs in the operative region before surgery. Animals were then placed in a stereotaxic apparatus and were under anesthesia after injection with sodium pentobarbital [30 mg/kg, intraperitoneally (i.p.)]. After disinfection of the operation areas, we performed an incision with sterile instruments (Figure 1A) to find the bregma (Figure 1B). ^125^I seeds were implanted and fixed with superglue on the skull surface 5 mm posterior relative to the bregma, 1 mm lateral to the sagittal suture, which corresponds to the middle part of the dorsal hippocampus. In the sham group, we implanted a titanium tube without the ^125^I nuclide core, whereas the other operation procedures were identical to the experimental groups. After 30 or 60 days of extracranial radiation, animals were perfused transcardially, and the skulls were carefully inspected for any shedding titanium tubes (Figures 1C,D). All tubes in the operated animals were retrieved in proper collection apparatus (Figure 1).

### Tissue Processing

For immunohistochemical experiments, animals were perfused via the ascending aorta with 4% paraformaldehyde in 0.01 M phosphate-buffered saline (pH 7.4, PBS) under overdose anesthesia (sodium pentobarbital, 100 mg/kg, i.p.). The whole brain was removed from the cranium and postfixed in the perfusion fixative for 2 days. The brains were transferred into solutions containing 15% and then 30% sucrose for cryoprotection. Before sectioning, a small portion of the surface cortex was removed from the contralateral cortex relative to the implanted side, for an orientation during microscopic examination (Figure 1I). The cerebrum was cut frontally in a cryostat at 30-μm thickness, with 24 sets of sections passing the rostrocaudal dimension of the hippocampus in all animals. The sections were collected serially in PBS in cell culture plates. Thus, each well contained equally distant (∼30 × 24 = 720 μm) sections from the septal to the temporo-occipital levels of the hippocampus in both hemispheres (Figure 1J).

### Immunohistochemistry

From each brain, separate sets of hippocampal sections were immunohistochemically stained with the avidin–biotin complex (ABC) method using previously characterized commercial primary antibodies, including Ki67, doublecortin (DCX), and neuron-specific nuclear antigen (NeuN) (Xiong et al., 2008; Yang et al., 2015; Wan et al., 2019). The sections were first treated in PBS containing 5% H_2_O_2_ for 30 min and in PBS containing 5% normal horse serum with 0.3% Triton X-100 for 2 h, to minimize non-specific labeling. The sections were then incubated with rabbit anti-Ki67 (Vector Laboratories, Burlingame, CA, United States; #014-1107, diluted at 1:1,000), goat anti-DCX (Santa Cruz Biotech.; sc-8066, 1:1,000), or NeuN (Abcam; #ab134014,1:2,000), overnight at 4°C with gentle agitation on a rotator. After three washes with PBS, the sections were incubated with biotinylated pan-specific secondary antibodies (horse anti-mouse, rabbit, and goat IgGs) at 1:400 for 2 h and ABC reagents (1:400) (Vector Laboratories) for another 2 h. The immunoreactive product was visualized with 0.003% H_2_O_2_ and 0.05% 3,3’-diaminobenzidine (DAB). We washed the sections three times with PBS for 10 min between the incubations. The immunolabeled sections were placed on glass microslides, dehydrated with ascending concentrations of ethanol, cleared with xylene, and mounted with coverslips using a mounting medium. The Ki67^+^ and DCX^+^ immunolabeled sections were also counterstained with hematoxylin for the histological examination under a microscope.

### Nissl Staining (Cresyl Violet Staining)

Sections were amounted onto gelatinized glass slides and air-dried at room temperature. Following several minutes in PBS, the sections were rinsed with distilled water and stained using a Nissl staining kit (1% cresyl violet staining, Solarbio, #G1430, China) for 60 min at 56°C in the dark. The samples were washed again with distilled water and differentiated in a Nissl differentiation solution until the background was light purple or colorless. The slides were dehydrated with 75, 80, and 90% ethanol sequentially for 2 min each, with absolute ethanol for 5 min two times, and with xylene for 10 min two times. They were then sealed with neutral gum and stored in the dark.

### Imaging, Cell Count, and Quantitative Analysis

The immunolabeled sections were examined initially on an Olympus BX51 microscope (CellSens Standard; Olympus Corporation, Japan) to validate the immunolabeling quality. All sections were scanned using the 20 × objective of a Motic-Olympus microscope equipped with an automated stage and imaging system (Wuhan, Hubei, China), which could yield a final auto-focused, montaged, and magnification-adjustable image covering the entire area of a glass slide.

### Quantification of Ki67^+^, DCX^+^ Cells in the Hippocampal Dentate Gyrus

Low-magnification (2× and 2.5×) and high-magnification (10×, 40×) images over the area of interest (AOI) were extracted from the images for figure illustration. For the sections immunolabeled with DAB as the chromogen, the entire area of hippocampal formation was imaged using 2× objective for orientation and measurement of the length of the granule cell layer (GCL) using the NIH ImageJ software. We then counted Ki67^+^, DCX^+^ cells along the subgranular zone (SGZ) in the dorsal dentate gyrus (DG) (Figure 1K). For all the measurements, the AOI was defined as a band region equal to the thickness of the GCL but centered over the SGZ (Figures 1L,M). Thus, we opened the images with Photoshop software and drew two lines along the upper and low blades of the GCL (Figure 1K). This lined template was then moved toward the hilus by half of the distance of the GCL thickness. After enlarging the images on the computer screen, we counted the numbers of Ki67^+^ and DCX^+^ cells and calculated the sums for each hippocampal section. The length of the GCL was calculated by referring to the scale bar. Finally, the cell density, expressed as the number of cells per unit (mm) length of GCL, was calculated for each brain, using the data obtained from the second to the fifth equally spaced (rostral-to-caudal direction, Figure 1J) hippocampal sections from both hemispheres (the first section at the septal end showed no or only a small cross-sectional area of the DG and was therefore not included in the quantification). We used the dorsal hippocampus for quantification to keep the sampling region consistent.

### Quantification of NeuN^+^ Cells in the Hippocampal Dentate Gyrus and CA1

Low-magnification (2.5×) and high-magnification (10×, 60×) images over the AOI were extracted from the images for figure illustration. These images were used to quantify the number of NeuN^+^ cells in the hippocampus. The GCL of the dorsal hippocampus and the stratum pyramidale (s.p.) in the CA1 region were randomly sampled using a method described recently (Ai et al., 2021). Thus, four or five 100 μm by 100 μm (10,000 μm^2^) squares in the GCL and the s.p were tagged. We then enlarged the images on the computer screen and counted the NeuN^+^ cells in the squares. The sums were calculated for each hippocampal section. The NeuN^+^ cell density, expressed as the number of cells per unit area (mm^2^) in the GCL and s.p., was calculated for each brain based on data obtained from the second to the fifth equally spaced (Figure 1J) sections from both hemispheres. As with the analysis of Ki67^+^ and DCX^+^ cells, we used the dorsal hippocampus for quantification to keep the sampling region consistent.

### Radiation Dosimetry

The dosage of radioactivity absorbed by the tissues from the initial source activity with different irradiation times was calculated with the following equation (Jones et al., 1995; Yu et al., 1999; Wang et al., 2017):

*D*_*r*_ = *S*_*k*_∧[*G*(*r*,θ_0_)/*G*(*r*_0_,θ_0_)]*g*(*r*)φ_*an*_(*r*)

In this equation, *S*_*k*_ represents the air kerma strength, φ_*an*_(r) is the ratio of the dose rate at distance *r*, *G* (*r*, θ_0_) represents the geometry factor, and *g*(*r*) is the radial dose function. The absorbed radioactive dosages at the reference point varied among the experimental groups with the initial source activity, the distance from the ^125^I seeds, and the follow-up times. In our research, we detected using a stereotactic instrument that the midpoint of the ^125^I seed radiation source was about 5.0 mm from the middle part of the ipsilateral hippocampus relative to the implanted side, and 3.25 mm from the sagittal suture of the skull. The levels of absorbed dosages in the hippocampus in regions located 5.0 and 8.2 mm transverse from the midpoint of the ^125^I seeds are summarized in Table 1.

**TABLE 1:** Levels of absorbed radioactive dosages in the hippocampus in regions located 5.0 and 8.2 mm from the ^125^I seeds.

**Radioactivity (mCi)**	**Absorbed radioactive dosage (ipsilateral hippocampus,**		**Absorbed radioactive dosage (contralateral hippocampus,**	
	**5 mm from ^125^I seeds, cGy)**		**8.2 mm from ^125^I seeds, cGy)**	
	**30 days**	**60 days**	**30 days**	**60 days**
0.8 mCi*3	–	12,847.60	–	6,881.40
1.0 mCi*1	3,110.00	5,353.17	1,727.2	2,893.65
0.6 mCi*1	1,866.00	3,211.89	1,090.32	1,790.18
0.3 mCi*1	933.00	1,605.95	598.16	955.09

### Data Assembly, Statistical Analysis, and Figure Preparation

Cell counting was performed by experimenters who were blinded to the animal grouping information, and the data were gathered by the primary experimenters for the quantitative and statistical analyses. The numeric density (number of cells/mm GCL length and number of cells/mm^2^ GCL and CA1 areas) were calculated in Excel spreadsheets and expressed as mean ± standard derivation (SD). We entered the data from individual animals into Prism spreadsheets (GraphPad Prism 5, San Diego, CA, United States) under the corresponding groups and generated graphs accordingly. Statistical analyses were performed to compare the means between the groups using one-way analysis of variance (ANOVA) tests together with Bonferroni multiple-comparisons *post hoc* tests. The figures were assembled with Photoshop 7.1 and converted into TIFF files after adjusting the contrast and brightness in the entire panel as needed.

## Results

The purpose of this study was to determine whether ^125^I seeds can effectively ablate newborn neurons and to also assess the degree of damage to mature neurons in the hippocampus. According to previous studies, the newborn neurons in adult guinea pigs reach their mature-like neuronal phenotype in approximately 60 days (Wan et al., 2019). In our preliminary experiments, we observed changes of DCX^+^ and NeuN^+^ neurons at 60 days after extracranial radiation with implantation of three 0.8-mCi ^125^I seeds. We also used Ki67 to identify the proliferating neural stem cells or neuroblasts (Scholzen and Gerdes, 2000; Xiong et al., 2008). Consistent with literature, Ki67^+^ cells were found along the SGZ of the DG, with the immunolabeling appeared as small nuclear profiles and tended to arrange in pairs or small clusters (Supplementary Figures 1A–C). DCX^+^ cells were also localized to the SGZ morphologically characteristic of immature neurons (Supplementary Figures 1J–L). Further, NeuN immunolabeling and Nissl stain were used to assess the damage of hippocampal mature neurons (Supplementary Figures 2, 3).

Based on the above preliminary experiments, brains in animals that suffered three 0.8-mCi local radiation for 60 days showed dramatic reduction of Ki67^+^ and DCX^+^ cells in both the ipsilateral and contralateral hippocampi relative to the sham group (Supplementary Figure 1). Thus, no Ki67^+^ and DCX^+^ cells could be detected in the ipsilateral GCL. NeuN labeling was also affected in the DG and CA1 in the radiated groups relative to control (Supplementary Figure 2). Specifically, a large number of dark brown granules occurred in the ipsilateral GCL and s.p. (Supplementary Figures 2K,L), whereas only a few were observed in the contralateral (Supplementary Figures 2G,H) side and none in the sham hippocampus. These dark brown granules may be produced by the destruction of neurons, likely the rupture of the nucleus after the high dose of ^125^I seed radiation. Based on the above observations, we concluded that, whereas the radiation effect caused by 3 × 0.8-mCi radiation for 60 days could effectively deplete hippocampal adult newborn neurons, the dosage was apparently too high, resulting in obvious radiotoxic effects on mature hippocampal neurons. In the formal experiments, ^125^I radiations at 1.0, 0.6, and 0.3 mCi were applied for 30 and 60 days postradiation. It should be noted that based on the clinical application of ^125^I seeds in radiation medicine, the minimum effective radioactivity to eliminate tumor proliferating cells is 0.3 mCi (Wang et al., 2017). Therefore, prescription-ready ^125^I tubes with 0.3 mCi radioactivity were used for the minimum dosage experiments in the current study.

### Assessment of the Spatial Range of ^125^I Radiation Effect on DCX^+^ Cells

**FIGURE 2:**
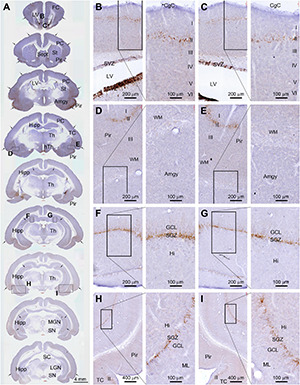
Topographic distribution of doublecortin immunoreactive (+) cells (with hematoxylin counterstain in blue) in normal adult (4-month-old) guinea pig cerebrum. **(A)** A set of low-magnification images of sections obtained from different rostrocaudal levels, with framed areas in different structures enlarged as the right panels as indicated **(B–I)**. DCX^+^ cells are located in the subventricular zone (SVZ) surrounding the lateral ventricle (LV) **(B,C)**, and along the SGZ of the dentate gyrus (DG) **(F–I)**. They are also distributed along layer II across the piriform cortex as well as neocortical regions **(A,D,E,H,I)**. The overall amount and distribution of DCX labeling around the LV, in the hippocampal formation, and across the cerebral cortex are present in a symmetric manner between the two hemispheres (as indicated by arrows in **A**). FC, frontal cortex; PC, parental cortex; TC, temporal cortex; CgC, cingulate cortex; Sept, septum; St, striatum; Pir, piriform cortex; Amyg, amygdalar complex; Hipp, hippocampal formation; Th, Thalamus; hTh, hypothalamus; MGN, medial geniculate nucleus; LGN, lateral geniculate nucleus; SN, substantia nigra; ML, molecular layer; GCL, granule cell layer; Hi, hilus; I-IV, layers I to VI. Scale bars are as indicated.

DCX^+^ labeling in neuronal somata and dendrites was microscopically prominent to observe (as compared to the Ki67 labeling in cell nuclei). Therefore, for an overall assessment of regional effect of radiation, we compared the amount of DCX^+^ labeling in the cerebrum across the rostrocaudal dimension and also between the two hemispheres in experimental animals relative to sham control. For reference, the distribution of DCX^+^ cells over the entire cerebrum is illustrated in Figure 1. Thus, as documented previously (Xiong et al., 2008, 2010), DCX^+^ cells occurred in the SVZ surrounding the lateral ventricle (LV) and the SGZ of the DG (Figures 2A–C,F–I), the two established principal sites of adult neurogenesis in mammalian brain. In addition, DCX^+^ cells were distributed along layer II across the piriform cortex, as well as neocortical regions (Figures 2A,D,E,H,I). It should be emphasized that the overall amount and distribution of DCX^+^ labeling around the LV, in the hippocampal formation, and across the cerebral cortex appeared in a symmetric manner between the two hemispheres, either viewed at low magnification (Figure 1A, arrows) or examined by closer observation (Figures 2B–I). Notably, different from primates and some non-primate species (Xiong et al., 2008; Cai et al., 2009; Zhang et al., 2009’ Piumatti et al., 2018; Ai et al., 2021), DCX^+^ immature neurons in the amygdala were not prominently present in guinea pigs (Figures 2A,D,E).

**FIGURE 3:**
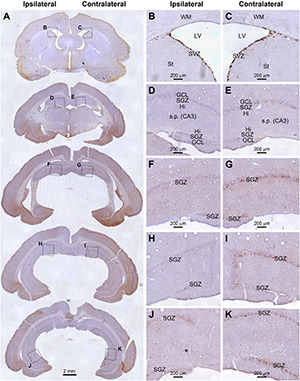
Reduced DCX immunolabeling in the hippocampus in an animal with unilateral extracranial ^125^I seed radiation at 0.6 mCi surviving 30 days. Panel arranges and scale bars are as indicated. The amount of immunolabeling in dentate gyrus (DG) are apparently asymmetric between the two hemispheres **(A–C)**. However, the labeling at the subventricular zone (SVZ) at the septal level appears comparable between the two sides. DCX labeling in the DG is greatly reduced in the ipsilateral side relative to the contralateral side from the rostral to caudal range of hippocampus over the dorsal and temporal subregions of the DG **(A,D–I)**. The reduction of DCX^+^ profiles in the most ventral part of the ipsilateral DG appeared less prominent relative to the contralateral counterpart **(A,J,K)**.

As illustrated, for example, in the cerebral sections from an animal with unilateral extracranial ^125^I seed radiation at 0.6 mCi surviving 30 days, the amount of DCX^+^ immunolabeling along the SGZ of the DG appeared dramatically asymmetric between the two hemispheres. However, the labeling at the SVZ at the septal level appeared largely comparable between the two sides (Figure 3). Specifically, DCX^+^ labeling was greatly reduced in the ipsilateral relative to the contralateral DG, which were evident essentially across the entire rostral to caudal range of the hippocampal formation. Further, the reduced labeling in the ipsilateral side could be clearly observed over the dorsal and temporal subregions of the DG over the entire rostrocaudal range of the hippocampus (Figures 3A,D,I). It should be noted that the reduction of DCX^+^ profiles in the most ventral part of the ipsilateral DG appeared less prominent relative to the contralateral counterpart (Figures 3A,J,K). Furthermore, in comparison with the sections from age-matched shame control (Figures 2A,F–I), it appeared that the overall amount of DCX^+^ labeling in the SGZ in the ipsilateral hippocampus from the radiated animal was also reduced to a certain degree (Figures 3A,G,I,K).

### Dosage Effect of ^125^I Radiation on Ki67^+^ Cells

**FIGURE 4:**
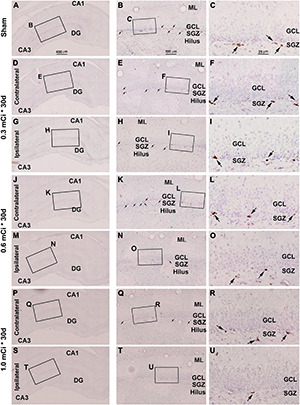
Effect of 0.3–1.0-mCi ^125^I seed radiation on Ki67-immunolabeled (+) cells of the hippocampal dentate gyrus at 30 days after implantation. Representative images of Ki67^+^ (brown) cells with hematoxylin counterstain (blue) from individual animal groups are illustrated as indicated. The left panels **(A,D,G,J,M,P,S)** are low-magnification images showing the orientation of Ammon’s horn (CA1, CA3) and dentate gyrus (DG), with framed areas enlarged as the middle panels **(B,E,H,K,N,Q,T)**, in which the boxed areas are further enlarged as the right panels **(C,F,I,L,O,R,U)**. Ki67^+^ nuclear profiles (pointed by arrows) are localized at the subgranular zone (SGZ), with some occurring in clusters **(C,F,I,L,O,R,U)**. The number of Ki67^+^ cells in the SGZ appears to be reduced in the radiated groups relative to the sham control, more evident in the ipsilateral side **(H,I,N,O,T,U)**. DG, dentate gyrus; ML, molecular layer; SGZ, subgranular zone; GCL, granule cell layer. Scale bar = 400 μm in **(A)** applying to **(D,G,J,M,P)**; equal to 100 μm for **(B,E,H,K,N,Q)**; and 25 μm for **(C,F,I,L,O,R)**.

**FIGURE 5:**
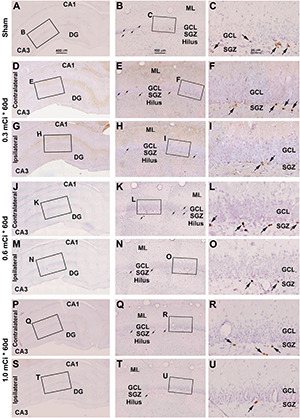
Effect of 0.3- to 1.0-mCi ^125^I seed radiation on Ki67^+^ cells of the hippocampal dentate gyrus at 60 days postsurgery. Representative images of Ki67 immunolabeling (brown) with hematoxylin counterstain (blue) from individual animal groups are as indicated. The left panels **(A,D,G,J,M,P,S)** are low-magnification images, with framed areas enlarged sequentially as the middle and right panels, as indicated. The amount of Ki67^+^ cells (pointed by arrows) in the SGZ is reduced in the radiation groups **(H,I,N,O,T,U)** relative to the sham control. Abbreviations are as defined in Figure 2. Scale bar = 400 μm in **(A)** applying to **(D,G,J,M,P)**; equal to 100 μm for **(B,E,H,K,N,Q)**; and 25 μm for **(C,F,I,L,O,R)**.

**FIGURE 6:**
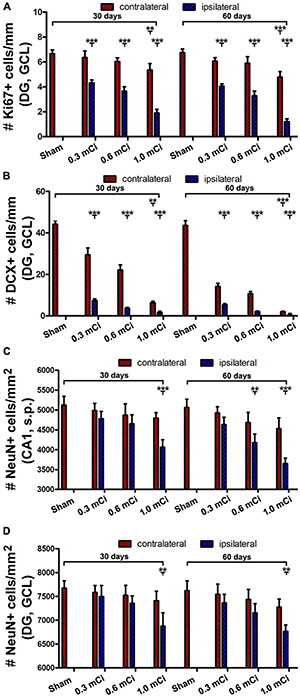
Quantification of Ki67, DCX^+^ and NeuN^+^ cells in ipsilateral and contralateral hippocampi in the radiated relative to sham control. The numeric densities of Ki67^+^ and DCX^+^ cells are expressed as number (#) of cells per mm of the GCL length of DG, NeuN^+^ cells, expressed as number (#) of cells per mm^2^ of the GCL and the stratum pyramidale (s.p.) of CA1 in the dorsal hippocampus in each groups. **(A,B)** Plot the mean densities of Ki67^+^ and DCX^+^ cells in the ipsilateral and contralateral sides in the sham group, and the groups with radiation after survival for 30 and 60 days, as indicated. **(C,D)** Illustrate the densities of NeuN^+^ cells in the GCL and the s.p. in CA1 in the control and radiation groups. Abbreviations are as defined in Figure 2. All data are represented as mean ± SD. Statistical analysis results are as indicated **p < 0.01 compared with sham, ***p < 0.001 compared with sham.

Microscopically, the overall amount of Ki67^+^ cells in the SGZ was reduced in all the radiation animal groups relative to sham control, which appeared more evident in the ipsilateral side in general (Figures 2, 3). This change was visible in the sections from animals subjected to ^125^I seed implantation surviving 30 days (Figure 4) as well as 60 days (Figure 5). The numeric densities, expressed as mean ± SD, of the subgranular Ki67^+^ cells in the dorsal hippocampus were measured for statistical analysis. The values in the animals surviving 30 days were 6.7 ± 0.7, 6.4 ± 0.6, 6.0 ± 0.6, 5.4 ± 0.5, 4.3 ± 0.5, 3.7 ± 0.4, and 1.9 ± 0.3 cells/mm of GCL length in the sham, 0.3 mCi-contral., 0.6 mCi-contral., 1.0 mCi-contral., 0.3 mCi-ipsil., 0.6 mCi-ipsil., and 1.0 mCi-ipsil. groups, respectively (Figure 6A). In the animals surviving 60 days, the numeric densities were 6.8 ± 0.6, 6.1 ± 0.7, 5.9 ± 0.5, 4.8 ± 0.7, 4.1 ± 0.2, 3.8 ± 0.7, and 1.2 ± 0.2 cells/mm in the sham, 0.3 mCi-contral., 0.6 mCi-contral., 1.0 mCi-contral., 0.3 mCi-ipsil., 0.6 mCi-ipsil., and 1.0 mCi-ipsil. groups, respectively (Figure 6A). Non-parametric tests (one-way ANOVA and Dunn multiple-comparisons tests) indicated that the mean densities were different among the different dosage groups surviving 30 and 60 days (30 days: *p* < 0.0001, *F* = 67.88; 60 days: *p* < 0.0001, *F* = 97.07). *Post hoc* tests reported a statistically significant difference between the sham group and the 1.0 mCi-contral., 0.3 mCi-ipsil., 0.6 mCi-ipsil., and 1.0 mCi-ipsil. groups at 30 and 60 days postsurgery, respectively (Figure 6A).

### Dosage Effect of ^125^I Radiation on DCX^+^ Cells

**FIGURE 7:**
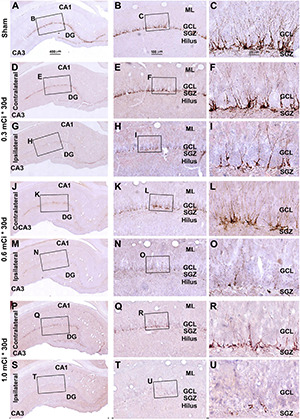
Characterization of hippocampal immature neurons with immunolabeling of doublecortin (DCX) in ipsilateral and contralateral hippocampal dentate gyrus relative to shamed guinea pigs after 0.3–1.0-mCi ^125^I seed radiation for 30 days. Representative images and enlarged areas of DCX immunolabeling (brown) with hematoxylin counterstain (blue) from individual animal groups are as indicated. The left panels are low-magnification images showing the orientation of Ammon’s horn (CA1, CA3) and dentate gyrus (DG), with framed areas enlarged as the middle and right panels, as indicated. DCX^+^ immature neurons are overwhelmingly localized to the SGZ, with labeled dendritic processes extending into the molecular layer (ML) **(C)**. DCX^+^ cells of the radiation groups are reduced bilaterally, more prominent in the ipsilateral side **(H,I,N,O,T,U)**, relative to the sham group, and show a decrease in the amount of dendritic labeling of the radiated groups **(H,I,N,O,T,U)**. Abbreviations are as defined in Figure 2. Scale bar = 400 μm in **(A)** applying to **(D,G,J,M,P,S)**; equal to 100 μm for **(B,E,H,K,N,Q,T)**; and 25 μm for **(C,F,I,L,O,R,U)**.

**FIGURE 8:**
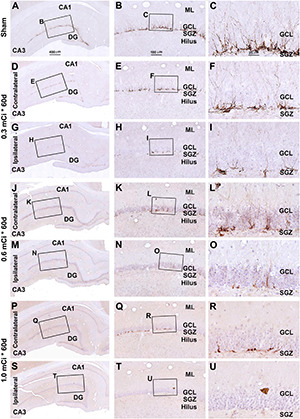
Characterization of DCX labeling in the ipsilateral and contralateral hippocampal dentate gyrus in animals with 0.3–1.0-mCi ^125^I seed radiation for 60 days relative to the sham control. Images of DCX immunolabeling (brown) with hematoxylin counterstain (blue) from representative animals are shown, with panel arrangements as indicated. DCX^+^ cells in the SGZ appear reduced in radiation groups relative to the sham group, as are their dendritic branches (right panels). Compared with Figure 4, the number and morphology changes of DCX^+^ cells in radiation group are more significant. Abbreviations are as defined in Figure 2. Scale bar = 400 μm in **(A)** applying to **(D,G,J,M,P,S)**; equal to 100 μm for **(B,E,H,K,N,Q,T)**; and 25 μm for **(C,F,I,L,O,R,U)**.

In microscopic examination, the overall amount of DCX-labeled profiles in the DG was overtly reduced in the sections from animals that received ^125^I radiation relative to sham controls, with this change appearing more dramatic in the ipsilateral side in reference to the implants (Figures 7, 8). At high magnification, the dendritic processes of DCX^+^ immature neurons also appeared fewer and shorter in radiated groups relative to sham controls and in the ipsilateral relative to contralateral hippocampi within the radiated groups (Figures 5B,C,E,F,H,I,K,L,N,O,Q,R,T,U, 7B,C,E,F, H,I,K,L,N,O,Q,R,T,U, 8B,C,E,F,H,I,K,L,N,O,Q,R,T,U). A dose-related impact on the morphology of subgranular DCX^+^ cells was also noticeable, such that the remaining labeled immature neurons appeared smaller, had few or no dendrites entering the molecular layer, and were more sparsely aligned along the SGZ in the higher dosage relative to lower dosage groups (Figures 7, 8, right panel images).

As with the aforementioned quantification of Ki67^+^ cells, we measured densities of the subgranular DCX^+^ cells per unit length of the GCL, given that their somata resided along the SGZ in a linear fashion. The densities of DCX^+^ cells were estimated to be 44.2 ± 4.9, 29.5 ± 3.3, 22.1 ± 2.5, 6.3 ± 0.7, 7.4 ± 0.8, 3.7 ± 0.4, and 1.7 ± 0.6 cells/mm in the sham, 0.3 mCi-contral., 0.6 mCi-contral., 1.0 mCi-contral., 0.3 mCi-ipsil., 0.6 mCi-ipsil., and 1.0 mCi-ipsil. groups surviving 30 days postsurgery, respectively. The values were 42.6 ± 4.5, 14.2 ± 1.5, 10.6 ± 1.1, 2.0 ± 0.2, 5.3 ± 0.6, 2.1 ± 0.2, and 0.4 ± 0.7 cells/mm in the sham, 0.3 mCi-contral., 0.6 mCi-contral., 1.0 mCi-contral., 0.3 mCi-ipsil., 0.6 mCi-ipsil., and 1.0 mCi-ipsil. groups at 60 days postsurgery, respectively. Statistically (one-way ANOVA), there existed significant differences of the means between the 30- and 60-day surviving groups (30 days: *p* < 0.0001, *F* = 157.7; 60 days: *p* < 0.0001, *F* = 252.3). *Post hoc* tests showed a significant difference of the means in the sham animal groups relative to individual radiated animal groups at both the 30- and 60-day surviving time points (Figure 6B).

### Dosage Effect of ^125^I Radiation on NeuN^+^ Neurons and Nissl-Stained Cells

We assessed potential radiation effect on NeuN^+^ cells and Nissl stain cells in the GCL of DG and the s.p. of CA1. Again, we chose the CA1 region of dorsal hippocampus for a consistent quantitative analysis, considering it is closer to the radiation site than CA3. We also analyzed in the GCL of dorsal DG for observational and quantitative analysis. As mentioned earlier, in our preliminary experiments, we noticed microscopically evident neurotoxic damage to mature hippocampal neurons based on NeuN and Nissl stain (Supplementary Figures 2, 3). We expected that lower radiation dosages used in the formal experiments would cause less or no apparent injurious effect on NeuN^+^ neurons and Nissl-stained cells.

**FIGURE 9:**
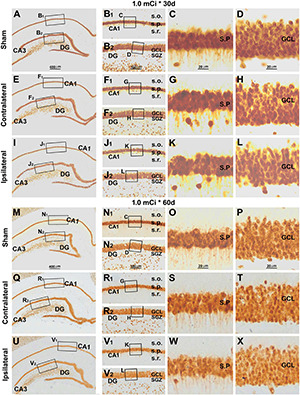
Lack of overt alteration in NeuN immunolabeling in the granule cell and pyramidal layers in animals with 1.0-mCi ^125^I seed radiation for 30 and 60 days relative to the sham control. The left panels are low-magnification images showing the orientation of Ammon’s horn (CA1, CA3) and dentate gyrus (DG), with framed areas enlarged sequentially as the middle and right panels, as indicated. NeuN-immunolabeled nuclei appear somewhat less densely packed in the GCL and the s.p. of CA1 in the radiated animals, more so in the 60-day surviving group (right panels). Abbreviations are as defined in Figures 2, 7. Scale bar = 400 μm in **(A)** applying to **(E,I,M,Q,U)**; equal to 100 μm for **(B1,B2,F1,F2,J1,J2,N1,N2,R1,R2,V1,V2)**; and 25 μm for **(C,D,G,H,K,L,O,P,S,T,W,X)**.

Indeed, there were no overt microscopic changes regarding the laminar organization and thickness of the CA1 region and DG while examining the NeuN and Nissl-stained sections from animals with 0. 3-, 0. 6-, and 1.0-mCi radiation surviving 30 and 60 days, relative to sham controls (Figure 9 and Supplementary Figures 4–7). Specifically, no disruption of cellular arrangement, irregularity of staining intensity, and nuclear breakage or condensation were visible in the NeuN^+^ neuron (Figure 9 and Supplementary Figures 4, 5) and Nissl-stained profiles (Supplementary Figures 6, 7) in the s.p. and GCL in both the ipsilateral and the contralateral sides in the radiated animals in comparison with controls. However, by closer examination, the NeuN^+^ nuclei in the s.p. and GCL appeared to be less densely packed in the radiated animals with 1.0-mCi treatment surviving 30 and 60 days, relative to sham control (Figures 9D,H,L,P,T,X).

Quantitatively, the numeric densities (mean ± SD, number of cells per square mm) of NeuN^+^ cells were 7,675.0 ± 153.5, 7,583.3 ± 145.7, 7,523.3 ± 206.5, 7,410.0 ± 200.8, 7,496.7 ± 231.6, 7,336.7 ± 220.1, and 6,876.7 ± 280.4 in the GCL in the sham, 0.3 mCi-contral., 0.6 mCi-contral., 1.0 mCi-contral., 0.3 mCi-ipsil., 0.6 mCi-ipsil., and 1.0 mCi-ipsil. groups surviving 30 days, respectively. The densities of the NeuN^+^ cells in the s.p. of CA1 were 5,123.3 ± 220.1, 4,986.7 ± 179.3, 4,870 ± 285.1, 4,690 ± 175.8, 4,780 ± 183.6, 4,653.3 ± 225.0, and 4,063.3 ± 190.4 cells/mm^2^ of s.p. area in the sham, 0.3 mCi-contral., 0.6 mCi-contral., 1.0 mCi-contral., 0.3 mCi-ipsil., 0.6 mCi-ipsil., and 1.0 mCi-ipsil. groups surviving 30 days, respectively. The density values for the 60-day surviving animals were 7,622.5 ± 204.2, 7,543.3 ± 214.6, 7,436.7 ± 309.9, 7,276.7 ± 165.0, 7,363.3 ± 180.1, 7,153.3 ± 195.0, and 6,767.6 ± 133.2 cells/mm^2^ in the GCL, and were 5,202.5 ± 212.2, 4,993.3 ± 211.3, 4,820 ± 262.1, 4,743.3 ± 215.0, 4,733.3 ± 181.5, 4,540 ± 251.2, and 3,903.3 ± 270.3 cells/mm^2^ in s.p. of CA1, in the sham, 0.3 mCi-contral., 0.6 mCi-contral., 1.0 mCi-contral., 0.3 mCi-ipsil., 0.6 mCi-ipsil., and 1.0 mCi-ipsil. groups, respectively. Statistically, there was a significant difference in the means between the groups at 30- and 60-day time points (CA1-30d: *p* = 0.0003, *F* = 8.94; DG-30d: *p* = 0.0003, *F* = 5.45; CA1-60d: *p* < 0.0001, *F* = 16.20; DG-60d: *p* = 0.0009, *F* = 7.29). *Post hoc* tests indicated a significant difference for the sham groups in comparison with the 0.6 mCi-ipsil. group of CA1-60d, the 1.0 mCi-ipsil. group of CA1-30d, DG-30d, CA1-60d, and DG-60d, whereas no difference existed for the remaining paired groups (Figures 6C,D).

### Extent of Local Cranial Trauma by ^125^I Radiation

**FIGURE 10:**
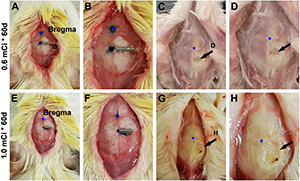
Minor skull trauma around the 0.6- and 1.0-mCi ^125^I seed after 60-day survival. The left panels **(A,E)** show the implantation location of the ^125^I seeds. **(B,F)** Are enlarged views of the implant areas. **(C,D,G,H)** Show the views after brain perfusion and the ^125^I seeds removed.

The current study used ^125^I isotope-filled tubes of the identical physical property but eliciting varying doses of radioactivity. The radioactivity was considered as the ultimate factor that led to the aforementioned changes in Ki67^+^ and DCX^+^ cells evidently in the hippocampal DG and, to a lesser extent, the changes noticeable in NeuN^+^ mature neurons and Nissl-stained profiles. By examining the extent of local tissue trauma (Lieberwirth et al., 2016), one could further cross-validate the existence of tissue injury that should be radioactivity-dependent. Therefore, we documented the cranial surface images over the implantation sites after animal perfusion and before brain removal. Overall, ^125^I seeds with lower radioactivity (e.g., 30 mCi), or shorter animal surviving time (e.g., the 30 days groups), were found to have milder local skull trauma (data not shown). Shown, for example (Figures 10A–D), in animals with 0.6-mCi radiation surviving 60 days, a fairly small and shallow depression (∼0.2 mm in diameter) of the cranial surface was found at the site of ^125^I tube implantation. In an animal with 1.0-mCi radiation surviving for 60 days, a larger and noticeably deeper skull depression (∼0.4 mm in diameter) was observed at the implantation sites (Figures 10E–H). It should be noted that in all animals included in the formal experiments in the current study, the skull damage did not penetrate the cranial bone (the parietal bone). However, in the animal with three 0.8-mCi seed implants that survived for 60 days, the skull damage was significant, with the cranial depression approximately 1.5 mm in size and the underneath brain tissue visible (Figure 1G).

## Discussion

### Extracranial ^125^I Radiation Produces Site- and Dose-Dependent Effect

^125^I is a radioisotope that emits γ rays with a low average photon energy of 28.5 keV, with a half-life of approximately 60 days (Krishnaswamy,1978). As elaborated in the Introduction, ^125^I seeds are commonly used in brachytherapy of many solid tumors (Schwarz et al., 2012; Wang et al., 2016; Zhang et al., 2016; Hirose et al., 2017; Brun et al., 2018; Vigneault et al., 2018; Zhu et al., 2019; Jia et al., 2020; Li et al., 2020; Watson et al., 2020). Given that radiation in general can result in inhibition or elimination of neurogenesis by blocking cell proliferation (Mizumatsu et al., 2003; Tan et al., 2011), we sort to establish a method to block AHN via localized extracranial application of ^125^I seeds using guinea pigs as experimental model. Guinea pigs are widely available and economically affordable laboratory animals. They have a larger brain than small laboratory rodents (e.g., mice and rats) and therefore could be better to allow the analysis of a space-related effect of radiation in the brain. As with mice and rats, DCX^+^ neuroblasts and immature neurons are present at the SVZ and SGZ relating to adult neurogenesis. DCX^+^ immature neurons are also distributed around layer II of the piriform cortex and the entire neocortex (Xiong et al., 2008; Bonfanti and Nacher, 2012; La Rosa et al., 2020). Therefore, this animal could be used to explore the response of different types of cerebral DCX^+^ cells to radiation (Xiong et al., 2010). The present study aimed to establish a “proof of principle” of a new and relatively simple approach for radioablation of adult neurogenesis in mammalian brain. Therefore, we focused on the hippocampal region and evaluated the effect by microscopic analysis of cellular profiles representing proliferative cells (Ki67) and developing/differentiating neurons (DCX) (Del Bigio, 1999; Kee et al., 2002). To identify an effective radiation dosage that can inhibit AHN yet yield limited damage to mature neuronal and non-neuronal tissue/cells, we designed multidosage experiments and also analyzed the hippocampal sections with NeuN immunolabeling and Nissl stain, as well as the skull regions around the ^125^I implants.

Our results show that by placing ^125^I seeds on the cranial area above the temporal lobe of the cerebrum, a region-selective blocking effect on HAN is achievable. Thus, DCX^+^ cells in the DG are apparently reduced microscopically across the entire rostrocaudal extent of the hippocampus. The loss of subgranular DCX^+^ neurons and processes are evident in the dorsal as well as temporal portions of the DG, whereas those in the ventral DG partially remained. The latter implicates a lesser impact of the extracranial radiation to adult neurogenesis in the deeper part of hippocampal formation. A distance-dependent effect is further conceivable by the fact that the loss of DCX^+^ cells is greater in the ipsilateral than the contralateral hippocampus in reference to the radiation source. It should be noted that the amount of DCX^+^ cells at the SVZ at the levels of the striatum appears to be largely symmetric between the two hemispheres, indicating a lesser impact of the temporal cranial radiation on subventricular adult neurogenesis at a cerebral level rostral to the hippocampal formation. It should be noted that unilateral cerebral X-radiation can simultaneously block neurogenesis in both the SVZ and SGZ in the ipsilateral hemisphere (Xiong et al., 2010). Taken together, our results indicate that localized extracranial ^125^I seed radiation can produce site-specific or space-limited inhibition of adult neurogenesis in guinea pig brain.

Our quantitative analysis of Ki67^+^ and DCX^+^ cells in the dorsal DG indicates that the effect of extracranial ^125^I radiation on HAN is dose-dependent. Thus, the extent of decrease in the densities of Ki67^+^ and DCX^+^ cells in the DG tends to increase as the radiation dosage rises from 0.3 mCi to 0.6 mCi, and to 1.0 mCi. This trend is seen in both the ipsilateral and contralateral hippocampi as compared to sham operation controls at the 30- and 60-day surviving time points. Hypothetically, one may expect that radiation effect on HAN should be also time-dependent. However, in the present study, the reduction in the densities of Ki67^+^ and DCX^+^ cells in the dorsal DG are already dramatic at the 30-day surviving time point, although a noticeable further decline is seen at the 60-day time point. This finding appears to suggest that a 30-day ^125^I radiation could yield a largely “saturated” or celling effect on the blockage of HAN. This result could be informative for the design of future studies using this method.

As mentioned in the Introduction, one concern for the use of radiation to block adult neurogenesis in the brain is the neurotoxic damage to mature neural components such as neurons, glia, and perhaps other cell types (e.g., vascular cells). This could be an issue involving confounding effect in functional studies on the role of adult neurogenesis. In our pilot experiment of this study, we did observe significant alterations in the morphology of NeuN^+^ neurons and Nissl-stained cells in the hippocampal formation in animals surviving 60 days with ∼2.4 mCi (3 × 0.8 mCi) radiation. However, our microscopic observation and quantitative analysis suggest that 30- and 60-day ^125^I radiations at 0.3–1.0 mCi appear to cause insignificant, if any, histological or neuronal damage even in the ipsilateral hippocampus. Specifically, neither quantitative nor quantitative changes are found in the NeuN^+^ neurons in the CA1 and DG in the 0.3- and 0.6-mCi groups surviving 30 and 60 days relative to sham controls. Accordingly, 0.3- and 0.6-mCi extracranial ^125^I radiations can apparently ablate HAN, but do not result in overt injurious impact on mature neuronal population.

### Extracranial ^125^I Radiation May Be Useful in Neurogenesis-Related Studies

Adult neurogenesis is a large topic in neuroscience, relating to basic mechanistic research, to pathogenesis of certain brain disorders, and to the development of therapeutic strategies for some neurological and psychological diseases (Shors et al., 2001; Cho et al., 2015; Radad et al., 2017; Fares et al., 2019; Abrous et al., 2021). Despite significant progress, much work is needed in each of the above frontiers. In general, ablation (knockout, knockdown or silencing in other terms, as used in genetic and molecular studies) is an important experimental approach to define the functional output of a particular biological system, from molecular to behavioral levels. The findings discussed above represent initial experimental data in support of a potential utility of localized extracranial ^125^I radiation in the investigation into HAN in mammalian brain. Besides the importance of extracranial ^125^I radiation leading to stereotactic and dose-dependent effect on adult neurogenesis elaborated previously, this method has other advantages on its application in animal experiments. For instance, the surgical procedure is rather simple and, with practice, can be completed shortly even under a brief inhalation anesthesia. This would cause fewer anesthesia-/operation-related animal mortalities and also minimize animal stress, which could be of importance in some experimental studies, such as that aimed to explore the functional implications of HAN during pregnancy, including the development of motherhood behavior in some mammals (Wan et al., 2019, 2021).

Different regions of the hippocampus appear to be engaged in different cognitive activities (Shors et al., 2002; Bannerman et al., 2004; Fanselow and Dong, 2010; Strange et al., 2014; Huckleberry et al., 2018; Trompoukis and Papatheodoropoulos, 2020). It is suggested that the dorsal hippocampus may perform primarily cognitive functions, whereas the ventral part is more related to the processing of stress, emotion and affect (Fanselow and Dong, 2010; Kheirbek and Hen, 2011). HAN in different regions of the longitudinal axis of the hippocampus may differentially participate in learning and memory and mode responses (Levone et al., 2021). At present, the ^125^I tubes were placed around the middle area of the temporal bone at the posterior part of the cranium. We see the radiation effect predominantly on the Ki67^+^ and DCX^+^ at the SGZ of the dorsal and temporal subregions of the DG in the ipsilateral side. These labeled cells were also reduced in the dorsal DG of the contralateral hippocampus relative to sham control according to cell count. The Ki67^+^ and DCX^+^ at the SGZ in the most ventral part of the DG appeared to be maintained largely. By adjusting the implant location and ^125^I dosage, it appears possible to allow the radiation effect projecting specifically or predominantly to the bilateral dorsal (e.g., a midline placement) hippocampus and temporal/ventral hippocampus (e.g., bilateral and lateral placement on the cranium, respectively). This type of surgical design could allow investigations into the role of HAN in different parts of the hippocampus in processing various cognitive and behavioral functions.

Another application of the extracranial ^125^I radiation method is to explore the extent, time course, and cellular feature of the recovery of HAN in mammalian brain, which may be also clinically relevant to radiation encephalopathy (Kickingereder et al., 2014; Weusthof et al., 2021; Zhang et al., 2021). The SGZ contains a pool of neural stem cells that are quiescent or undergoing proliferation, with the latter event leading to both neuronal and glial genesis (Del Bigio, 1999; Fares et al., 2019; Gage, 2019). However, less clear is whether, and if so, the extent of which, radiation would impact the populations of neural stems, the neuronal and glial fates of the precursor cells, and the time course and degree of neurogenesis recovery (Jinno, 2011; Kohler et al., 2011; Amrein, 2015). The relatively long lifespan of guinea pigs might allow a follow-up of morphological and behavioral recovery over an extended time, if needed.

In summary, we explored the feasibility of using extracranial ^125^I seed implantation to block hippocampal adult neurogenesis using guinea pigs as animal model, and cell proliferation (Ki67) and immature neuronal (DCX) markers as cause–effect indexers. Observational and quantitative analyses suggest that this approach can lead to stereotactic radioablation of hippocampal adult neurogenesis without dramatically damaging the mature neuronal populations. This method may be optimized to explore the functions of adult neurogenesis in mammalian brain.

## Data Availability Statement

The original contributions presented in the study are included in the article/Supplementary Material, further inquiries can be directed to the corresponding author/s.

## Ethics Statement

The animal study was reviewed and approved by the Ethics Review Committee of Xiangya Hospital, numbers 201603296 and 201603297.

## Author Contributions

LW, Z-HL, YZ, and JL were responsible for animal experimentation, tissue processing, immunohistochemistry, imaging acquisition, and statistical analyses. R-JH, CY, J-QA, and QZ were responsible for cell counting. LW drafted and revised the manuscript. X-XY revised and finalized the manuscript. J-EG, ET, AP, X-XY, and BX provided financial support. All authors contributed to the article and approved the submitted version.

## Conflict of Interest

The authors declare that the research was conducted in the absence of any commercial or financial relationships that could be construed as a potential conflict of interest.

## Publisher’s Note

All claims expressed in this article are solely those of the authors and do not necessarily represent those of their affiliated organizations, or those of the publisher, the editors and the reviewers. Any product that may be evaluated in this article, or claim that may be made by its manufacturer, is not guaranteed or endorsed by the publisher.

## References

[B1] Abrous D. N. Koehl M. Lemoine M. (2021). A baldwin interpretation of adult hippocampal neurogenesis: from functional relevance to physiopathology. *Mol. Psychiatry.* 10.1038/s41380-021-01172-4 [Epub ahead of print] 34103674 PMC8960398

[B2] Ai J. Q. Luo R. Tu T. Yang C. Jiang J. Zhang B. (2021). Doublecortin-expressing neurons in chinese tree shrew forebrain exhibit mixed rodent and primate-like topographic characteristics. *Front. Neuroanat* 15:727883. 10.3389/fnana.2021.727883 PMC8481370 34602987

[B3] Aimone J. B. Wiles J. Gage F. H. (2006). Potential role for adult neurogenesis in the encoding of time in new memories. *Nat. Neurosci.* 9 723–727. 10.1038/nn1707 16732202

[B4] Alam M. J. Kitamura T. Saitoh Y. Ohkawa N. Kondo T. Inokuchi K. (2018). Adult neurogenesis conserves hippocampal memory capacity. *J. Neurosci.* 38 6854–6863. 10.1523/jneurosci.2976-17.2018 29986876 PMC6596118

[B5] Altman J. (1963). Autoradiographic investigation of cell proliferation in the brains of rats and cats. *Anat. Rec.* 145 573–591. 10.1002/ar.1091450409 14012334

[B6] Altman J. Das G. D. (1965). Post-natal origin of microneurones in the rat brain. *Nature* 207 953–956. 10.1038/207953a0 5886931

[B7] Amrein I. (2015). Adult hippocampal neurogenesis in natural populations of mammals. *Cold Spring Harb Perspect. Biol.* 7:a021295. 10.1101/cshperspect.a021295 25934014 PMC4448614

[B8] Amrein I. Isler K. Lipp H. P. (2011). Comparing adult hippocampal neurogenesis in mammalian species and orders: influence of chronological age and life history stage. *Eur. J. Neurosci.* 34 978–987. 10.1111/j.1460-9568.2011.07804.x 21929629

[B9] Anacker C. Hen R. (2017). Adult hippocampal neurogenesis and cognitive flexibility - linking memory and mood. *Nat. Rev. Neurosci.* 18 335–346. 10.1038/nrn.2017.45 28469276 PMC6261347

[B10] Bannerman D. M. Rawlins J. N. McHugh S. B. Deacon R. M. Yee B. K. Bast T. (2004). Regional dissociations within the hippocampus–memory and anxiety. *Neurosci. Biobehav. Rev.* 28 273–283. 10.1016/j.neubiorev.2004.03.004 15225971

[B11] Ben Abdallah N. M. Filipkowski R. K. Pruschy M. Jaholkowski P. Winkler J. Kaczmarek L. (2013). Impaired long-term memory retention: common denominator for acutely or genetically reduced hippocampal neurogenesis in adult mice. *Behav. Brain Res.* 252 275–286. 10.1016/j.bbr.2013.05.034 23714078

[B12] Berdugo-Vega G. Arias-Gil G. López-Fernández A. Artegiani B. Wasielewska J. M. Lee C. C. (2020). Increasing neurogenesis refines hippocampal activity rejuvenating navigational learning strategies and contextual memory throughout life. *Nat. Commun.* 11:135. 10.1038/s41467-019-14026-z 31919362 PMC6952376

[B13] Bonfanti L. Nacher J. (2012). New scenarios for neuronal structural plasticity in non-neurogenic brain parenchyma: the case of cortical layer II immature neurons. *Prog. Neurobiol.* 98 1–15. 22609484 10.1016/j.pneurobio.2012.05.002

[B14] Brun T. Ferron G. Filleron T. Bonnet J. Martinez A. Ducassou A. (2018). Experimental study of pelvic perioperative brachytherapy with iodine 125 seeds (I-125) in an animal model. *J. Contemp Brachyther.* 10 463–469. 10.5114/jcb.2018.79470 30479624 PMC6251451

[B15] Bulin S. E. Mendoza M. L. Richardson D. R. Song K. H. Solberg T. D. Yun S. (2018). Dentate gyrus neurogenesis ablation via cranial irradiation enhances morphine self-administration and locomotor sensitization. *Addict. Biol.* 23 665–675. 10.1111/adb.12524 28626932 PMC5775053

[B16] Cai Y. Xiong K. Chu Y. Luo D. W. Luo X. G. Yuan X. Y. (2009). Doublecortin expression in adult cat and primate cerebral cortex relates to immature neurons that develop into GABAergic subgroups. *Exp. Neurol.* 216 342–356. 10.1016/j.expneurol.2008.12.008 19166833 PMC2902881

[B17] Cho K. O. Lybrand Z. R. Ito N. Brulet R. Tafacory F. Zhang L. (2015). Aberrant hippocampal neurogenesis contributes to epilepsy and associated cognitive decline. *Nat. Commun.* 6:6606. 10.1038/ncomms7606 25808087 PMC4375780

[B18] Coras R. Siebzehnrubl F. A. Pauli E. Huttner H. B. Njunting M. Kobow K. (2010). Low proliferation and differentiation capacities of adult hippocampal stem cells correlate with memory dysfunction in humans. *Brain* 133 3359–3372. 10.1093/brain/awq215 20719879

[B19] Del Bigio M. R. (1999). Proliferative status of cells in adult human dentate gyrus. *Microsci. Res. Tech.* 45 353–358. 10.1002/(SICI)1097-0029(19990615)45:6<353::AID-JEMT3<3.0.CO;2-M 10402263

[B20] Fanselow M. S. Dong H. W. (2010). Are the dorsal and ventral hippocampus functionally distinct structures? *Neuron* 65 7–19. 10.1016/j.neuron.2009.11.031 20152109 PMC2822727

[B21] Fares J. Bou Diab Z. Nabha S. Fares Y. (2019). Neurogenesis in the adult hippocampus: history, regulation, and prospective roles. *Int. J. Neurosci.* 129 598–611. 10.1080/00207454.2018.1545771 30433866

[B22] Gage F. H. (2019). Adult neurogenesis in mammals. *Science* 364 827–828. 10.1126/science.aav6885 31147506

[B23] Hirose K. Aoki M. Sato M. Akimoto H. Hashimoto Y. Imai A. (2017). Analysis of the relationship between prescribed dose and dosimetric advantage of real-time intraoperatively built custom-linked seeds in iodine-125 prostate brachytherapy. *Radiat Oncol.* 12:192. 10.1186/s13014-017-0932-7 29191234 PMC5710060

[B24] Houben S. Leroy K. Ando K. Yilmaz Z. Widomski C. Buée L. (2019). Genetic ablation of tau in postnatal neurons rescues decreased adult hippocampal neurogenesis in a tauopathy model. *Neurobiol. Dis.* 127 131–141. 10.1016/j.nbd.2019.02.021 30818066

[B25] Huckleberry K. A. Shue F. Copeland T. Chitwood R. A. Yin W. Drew M. R. (2018). Dorsal and ventral hippocampal adult-born neurons contribute to context fear memory. *Neuropsychopharmacology* 43 2487–2496. 29941977 10.1038/s41386-018-0109-6 PMC6180107

[B26] Jia S. N. Wen F. X. Gong T. T. Li X. Wang H. J. Sun Y. M. (2020). A review on the efficacy and safety of iodine-125 seed implantation in unresectable pancreatic cancers. *Int. J. Radiat. Biol.* 96 383–389. 10.1080/09553002.2020.1704300 31977258

[B27] Jinno S. (2011). Decline in adult neurogenesis during aging follows a topographic pattern in the mouse hippocampus. *J. Comput. Neurol.* 519 451–466. 10.1002/cne.22527 21192078

[B28] Jones D. Washington J. T. Christopherson D. A. (1995). Comments on “dosimetry of interstitial brachytherapy sources: recommendations of the AAPM radiation therapy committee task group no. 43”. *Med. Phys.* 22:1351. 10.1118/1.597521 7476725

[B29] Kang E. Wen Z. Song H. Christian K. M. Ming G. L. (2016). Adult neurogenesis and psychiatric disorders. *Cold Spring Harb Perspect. Biol.* 8:a019026. 10.1101/cshperspect.a019026 26801682 PMC5008067

[B30] Kee N. Sivalingam S. Boonstra R. Wojtowicz J. M. (2002). The utility of Ki-67 and BrdU as proliferative markers of adult neurogenesis. *J. Neurosci. Methods* 115 97–105. 10.1016/s0165-0270(02)00007-9 11897369

[B31] Kheirbek M. A. Hen R. (2011). Dorsal vs ventral hippocampal neurogenesis: implications for cognition and mood. *Neuropsychopharmacology* 36 373–374. 10.1038/npp.2010.148 21116266 PMC3055508

[B32] Kickingereder P. Hamisch C. Suchorska B. Galldiks N. Visser-Vandewalle V. Goldbrunner R. (2014). Low-dose rate stereotactic iodine-125 brachytherapy for the treatment of inoperable primary and recurrent glioblastoma: single-center experience with 201 cases. *J. Neurooncol.* 120 615–623. 10.1007/s11060-014-1595-y 25151509

[B33] Kohler S. J. Williams N. I. Stanton G. B. Cameron J. L. Greenough W. T. (2011). Maturation time of new granule cells in the dentate gyrus of adult macaque monkeys exceeds six months. *Proc. Natl. Acad. Sci. U.S.A.* 108 10326–10331. 10.1073/pnas.1017099108 21646517 PMC3121825

[B34] Kostin A. Alam M. A. McGinty D. Szymusiak R. Alam M. N. (2019). Chronic suppression of hypothalamic cell proliferation and neurogenesis induces aging-like changes in sleep-wake organization in young mice. *Neuroscience* 404 541–556. 10.1016/j.neuroscience.2019.01.053 30738854

[B35] Kuhn H. G. Toda T. Gage F. H. (2018). Adult hippocampal neurogenesis: a coming-of-age story. *J. Neurosci.* 38 10401–10410. 10.1523/jneurosci.2144-18.2018 30381404 PMC6284110

[B36] La Rosa C. Cavallo F. Pecora A. Chincarini M. Ala U. Faulkes C. G. (2020). Phylogenetic variation in cortical layer II immature neuron reservoir of mammals. *Elife* 9:e55456. 10.7554/eLife.55456 PMC7373429 32690132

[B37] Levone B. R. Codagnone M. G. Moloney G. M. Nolan Y. M. Cryan J. F. O’ Leary O. F. (2021). Adult-born neurons from the dorsal, intermediate, and ventral regions of the longitudinal axis of the hippocampus exhibit differential sensitivity to glucocorticoids. *Mol. Psychiatry* 26 3240–3252. 10.1038/s41380-020-0848-8 32709996

[B38] Li H. Duan Z. Zhao C. Fang W. Jia Y. Li X. (2020). Combination of brachytherapy with iodine-125 seeds and systemic chemotherapy versus systemic chemotherapy alone for synchronous extracranial oligometastatic non-small cell lung cancer. *Cancer Manag. Res.* 12 8209–8220. 10.2147/cmar.S267694 32982417 PMC7494957

[B39] Lieberwirth C. Pan Y. Liu Y. Zhang Z. Wang Z. (2016). Hippocampal adult neurogenesis: its regulation and potential role in spatial learning and memory. *Brain Res.* 1644 127–140. 10.1016/j.brainres.2016.05.015 27174001 PMC5064285

[B40] McDonald H. Y. Wojtowicz J. M. (2005). Dynamics of neurogenesis in the dentate gyrus of adult rats. *Neurosci. Lett.* 385 70–75. 10.1016/j.neulet.2005.05.022 15967575

[B41] Micheli L. Ceccarelli M. D’Andrea G. Tirone F. (2018). Depression and adult neurogenesis: positive effects of the antidepressant fluoxetine and of physical exercise. *Brain Res. Bull.* 143 181–193. 10.1016/j.brainresbull.2018.09.002 30236533

[B42] Miller S. M. Sahay A. (2019). Functions of adult-born neurons in hippocampal memory interference and indexing. *Nat. Neurosci.* 22 1565–1575. 10.1038/s41593-019-0484-2 31477897 PMC7397477

[B43] Mizumatsu S. Monje M. L. Morhardt D. R. Rola R. Palmer T. D. Fike J. R. (2003). Extreme sensitivity of adult neurogenesis to low doses of X-irradiation. *Cancer Res.* 63 4021–4027. 12874001

[B44] Peng S. Yang B. Duan M. Y. Liu Z. W. Wang W. F. Zhang X. Z. (2019). The disparity of impairment of neurogenesis and cognition after acute or fractionated radiation exposure in adolescent BALB/c mice. *Dose Res.* 17:574. 10.1177/1559325818822574 30670940 PMC6327339

[B45] Piumatti M. Palazzo O. La Rosa C. Crociara P. Parolisi R. Luzzati F. (2018). Non-newly generated, “immature” neurons in the sheep brain are not restricted to cerebral cortex. *J. Neurosci.* 38 826–842. 10.1523/jneurosci.1781-17.2017 29217680 PMC6596233

[B46] Radad K. Moldzio R. Al-Shraim M. Kranner B. Krewenka C. Rausch W. D. (2017). Recent advances on the role of neurogenesis in the adult brain: therapeutic potential in Parkinson’s and Alzheimer’s diseases. *CNS Neurol. Disord Drug Targets* 16 740–748. 10.2174/1871527316666170623094728 28641510

[B47] Rivera P. D. Simmons S. J. Reynolds R. P. Just A. L. Birnbaum S. G. Eisch A. J. (2019). Image-guided cranial irradiation-induced ablation of dentate gyrus neurogenesis impairs extinction of recent morphine reward memories. *Hippocampus* 29 726–735. 10.1002/hipo.23071 30779299 PMC7036142

[B48] Saxe M. D. Battaglia F. Wang J. W. Malleret G. David D. J. Monckton J. E. (2006). Ablation of hippocampal neurogenesis impairs contextual fear conditioning and synaptic plasticity in the dentate gyrus. *Proc. Natl. Acad. Sci. U.S.A.* 103 17501–17506. 10.1073/pnas.0607207103 17088541 PMC1859958

[B49] Scholzen T. Gerdes J. (2000). The Ki-67 protein: from the known and the unknown. *J. Cell Physiol.* 182 311–322. 10.1002/(SICI)1097-4652(200003)182:3<311::AID-JCP1<3.0.CO;2-9 10653597

[B50] Schwarz S. B. Thon N. Nikolajek K. Niyazi M. Tonn J. C. Belka C. (2012). Iodine-125 brachytherapy for brain tumours–a review. *Radiat Oncol.* 7:30. 10.1186/1748-717x-7-30 22394548 PMC3354996

[B51] Shors T. J. Miesegaes G. Beylin A. Zhao M. Rydel T. Gould E. (2001). Neurogenesis in the adult is involved in the formation of trace memories. *Nature* 410 372–376. 10.1038/35066584 11268214

[B52] Shors T. J. Townsend D. A. Zhao M. Kozorovitskiy Y. Gould E. (2002). Neurogenesis may relate to some but not all types of hippocampal-dependent learning. *Hippocampus* 12 578–584. 10.1002/hipo.10103 12440573 PMC3289536

[B53] Strange B. A. Witter M. P. Lein E. S. Moser E. I. (2014). Functional organization of the hippocampal longitudinal axis. *Nat. Rev. Neurosci.* 15 655–669. 10.1038/nrn3785 25234264

[B54] Surget A. Belzung C. (2021). Adult hippocampal neurogenesis shapes adaptation and improves stress response: a mechanistic and integrative perspective. *Mol. Psychiatry* 10.1038/s41380-021-01136-8 [Epub ahead of print] 33990771 PMC8960391

[B55] Tada E. Parent J. M. Lowenstein D. H. Fike J. R. (2000). X-irradiation causes a prolonged reduction in cell proliferation in the dentate gyrus of adult rats. *Neuroscience* 99 33–41. 10.1016/s0306-4522(00)00151-2 10924950

[B56] Tan Y. F. Rosenzweig S. Jaffray D. Wojtowicz J. M. (2011). Depletion of new neurons by image guided irradiation. *Front. Neurosci.* 5:59. 10.3389/fnins.2011.00059 21541259 PMC3083759

[B57] Tang F. R. Loke W. K. Wong P. Khoo B. C. (2017). Radioprotective effect of ursolic acid in radiation-induced impairment of neurogenesis, learning and memory in adolescent BALB/c mouse. *Physiol. Behav.* 175 37–46. 10.1016/j.physbeh.2017.03.027 28341234

[B58] Toda T. Gage F. H. (2018). Review: adult neurogenesis contributes to hippocampal plasticity. *Cell Tissue Res.* 373 693–709. 10.1007/s00441-017-2735-4 29185071

[B59] Toda T. Parylak S. L. Linker S. B. Gage F. H. (2019). The role of adult hippocampal neurogenesis in brain health and disease. *Mol. Psychiatry* 24 67–87. 10.1038/s41380-018-0036-2 29679070 PMC6195869

[B60] Trompoukis G. Papatheodoropoulos C. (2020). Dorsal-ventral differences in modulation of synaptic transmission in the hippocampus. *Front. Synaptic Neurosci.* 12:24. 10.3389/fnsyn.2020.00024 32625076 PMC7316154

[B61] Vigneault E. Martell K. Taussky D. Husain S. Delouya G. Mbodji K. (2018). Does seed migration increase the risk of second malignancies in prostate cancer patients treated with iodine-125 loose seeds brachytherapy? *Int. J. Radiat Oncol. Biol. Phys.* 100 1190–1194. 10.1016/j.ijrobp.2017.12.273 29428250

[B62] Wan L. Huang R. J. Luo Z. H. Gong J. E. Pan A. Manavis J. (2021). Reproduction-associated hormones and adult hippocampal neurogenesis. *Neural Plast.* 2021:3651735. 10.1155/2021/3651735 34539776 PMC8448607

[B63] Wan L. Tu T. Zhang Q. L. Jiang J. Yan X. X. (2019). Pregnancy promotes maternal hippocampal neurogenesis in guinea pigs. *Neural. Plast* 2019:5765284. 10.1155/2019/5765284 31097956 PMC6487096

[B64] Wang C. Chen Z. Sun W. Yasin Y. Zhang C. Ma X. (2016). Palliative treatment of pelvic bone tumors using radioiodine ((125) I) brachytherapy. *World J. Surg. Oncol.* 14:294. 10.1186/s12957-016-1050-y 27884196 PMC5123313

[B65] Wang Y. Guo J. H. Zhu G. Y. Zhu H. D. Chen L. Lu J. (2017). A novel self-expandable, radioactive airway stent loaded with (125) I seeds: a feasibility and safety study in healthy beagle dog. *Cardiovasc. Intervent. Radiol.* 40 1086–1093. 10.1007/s00270-017-1639-8 28389860

[B66] Watson J. Romagna A. Ballhausen H. Niyazi M. Lietke S. Siller S. (2020). Long-term outcome of stereotactic brachytherapy with temporary Iodine-125 seeds in patients with WHO grade II gliomas. *Radiat Oncol.* 15:275. 10.1186/s13014-020-01719-9 33298103 PMC7724805

[B67] Weusthof K. Lüttich P. Regnery S. König L. Bernhardt D. Witt O. (2021). Neurocognitive outcomes in pediatric patients following brain irradiation. *Cancers (Basel)* 13:3538. 10.3390/cancers13143538 34298751 PMC8307409

[B68] Wojtowicz J. M. (2006). Irradiation as an experimental tool in studies of adult neurogenesis. *Hippocampus* 16 261–266. 10.1002/hipo.20158 16435311

[B69] Xiong K. Cai Y. Zhang X. M. Huang J. F. Liu Z. Y. Fu G. M. (2010). Layer I as a putative neurogenic niche in young adult guinea pig cerebrum. *Mol. Cell. Neurosci.* 45 180–191. 10.1016/j.mcn.2010.06.009 20599617 PMC2923265

[B70] Xiong K. Luo D. W. Patrylo P. R. Luo X. G. Struble R. G. Clough R. W. (2008). Doublecortin-expressing cells are present in layer II across the adult guinea pig cerebral cortex: partial colocalization with mature interneuron markers. *Exp. Neurol.* 211 271–282. 10.1016/j.expneurol.2008.02.003 18378231 PMC2994188

[B71] Yang Y. Xie M. X. Li J. M. Hu X. Patrylo P. R. Luo X. G. (2015). Prenatal genesis of layer II doublecortin expressing neurons in neonatal and young adult guinea pig cerebral cortex. *Front. Neuroanat* 9:109. 10.3389/fnana.2015.00109 PMC4530311 26321922

[B72] Yeung S. T. Myczek K. Kang A. P. Chabrier M. A. Baglietto-Vargas D. Laferla F. M. (2014). Impact of hippocampal neuronal ablation on neurogenesis and cognition in the aged brain. *Neuroscience* 259 214–222. 10.1016/j.neuroscience.2013.11.054 24316470 PMC4438704

[B73] Youssef M. Krish V. S. Kirshenbaum G. S. Atsak P. Lass T. J. Lieberman S. R. (2018). Ablation of proliferating neural stem cells during early life is sufficient to reduce adult hippocampal neurogenesis. *Hippocampus* 28 586–601. 10.1002/hipo.22962 29742815 PMC6167166

[B74] Yu Y. Anderson L. L. Li Z. Mellenberg D. E. Nath R. Schell M. C. (1999). Permanent prostate seed implant brachytherapy: report of the American association of physicists in medicine task group no. 64. *Med. Phys.* 26 2054–2076. 10.1118/1.598721 10535622

[B75] Zhang W. Luo J. Liu Q. Ma J. Qu X. Yang M. (2016). Brachytherapy with Iodine-125 seeds strand for treatment of main portal vein tumor thrombi: an experimental study in a rabbit model. *Am. J. Cancer Res.* 6 587–599. 27152237 PMC4851839

[B76] Zhang X. M. Cai Y. Chu Y. Chen E. Y. Feng J. C. Luo X. G. (2009). Doublecortin-expressing cells persist in the associative cerebral cortex and amygdala in aged nonhuman primates. *Front. Neuroanat.* 3:17. 10.3389/neuro.05.017.2009 19862344 PMC2766270

[B77] Zhang Y. M. Kang Y. F. Zeng J. J. Li L. Gao J. M. Liu L. Z. (2021). Surface-based falff: a potential novel biomarker for prediction of radiation encephalopathy in patients with nasopharyngeal carcinoma. *Front. Neurosci.* 15:692575. 10.3389/fnins.2021.692575 34349618 PMC8326829

[B78] Zhu Y. Dong M. Yang J. Zhang J. (2019). Evaluation of iodine-125 interstitial brachytherapy using micro-positron emission tomography/computed tomography with 18F-fluorodeoxyglucose in hepatocellular carcinoma hepg2 xenografts. *Med. Sci. Monit.* 25 371–380. 10.12659/msm.912590 30636171 PMC6339452

