# Supplementary material for: Extracranial 125I Seed Implantation Allows Non-invasive Stereotactic Radioablation of Hippocampal Adult Neurogenesis in Guinea Pigs
Source: Front Neurosci. 2021 Nov 30;15:756658. doi: 10.3389/fnins.2021.756658 (PMC8670234; doi:10.3389/fnins.2021.756658)
Supplement: Supplementary file 1 [file Data_Sheet_1.pdf]

Supplemental Figures for Manuscript #756658

Extracranial ^{125}I seed implantation allows non-invasive stereotactic radioablation of hippocampal adult neurogenesis in guinea pigs

Lily Wan[1,2], Rou-Jie Huang[3], Chen Yang[2], Jia-Qi Ai[2], Qian Zhou[3], Jiao-E Gong[4], Jian Li[5], Yun Zhang[6], Zhao-Hui Luo[1], Ewen Tu[7], Aihua Pan[2], Bo Xiao[1]*, and Xiao-Xin Yan[2]*

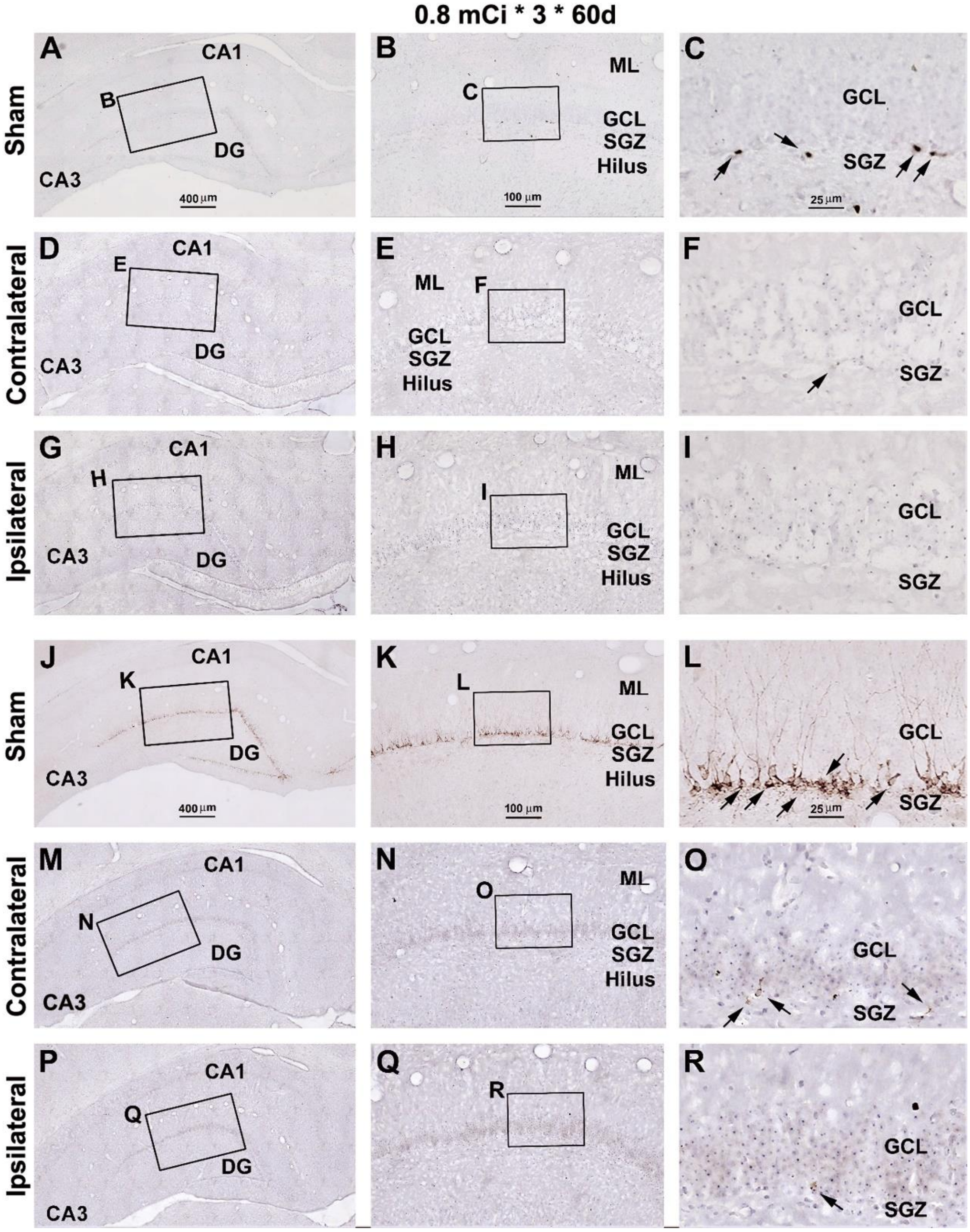

Supplemental Figure 1: Ki67 and doublecortin (DCX) immunolabeling in hippocampal formation with 0.8 mCi radiation for 60 days relative to the sham control. Panels (A-I) are Ki67 immunolabeling images from sham control and radiated animals as indicated. Panels (J-R) are DCX immunolabeling images from sham control and radiated animals as indicated. There is a near-complete loss of the Ki67 and DCX labeling in the radiated hippocampi relative to control as clearly seen in the right panels.

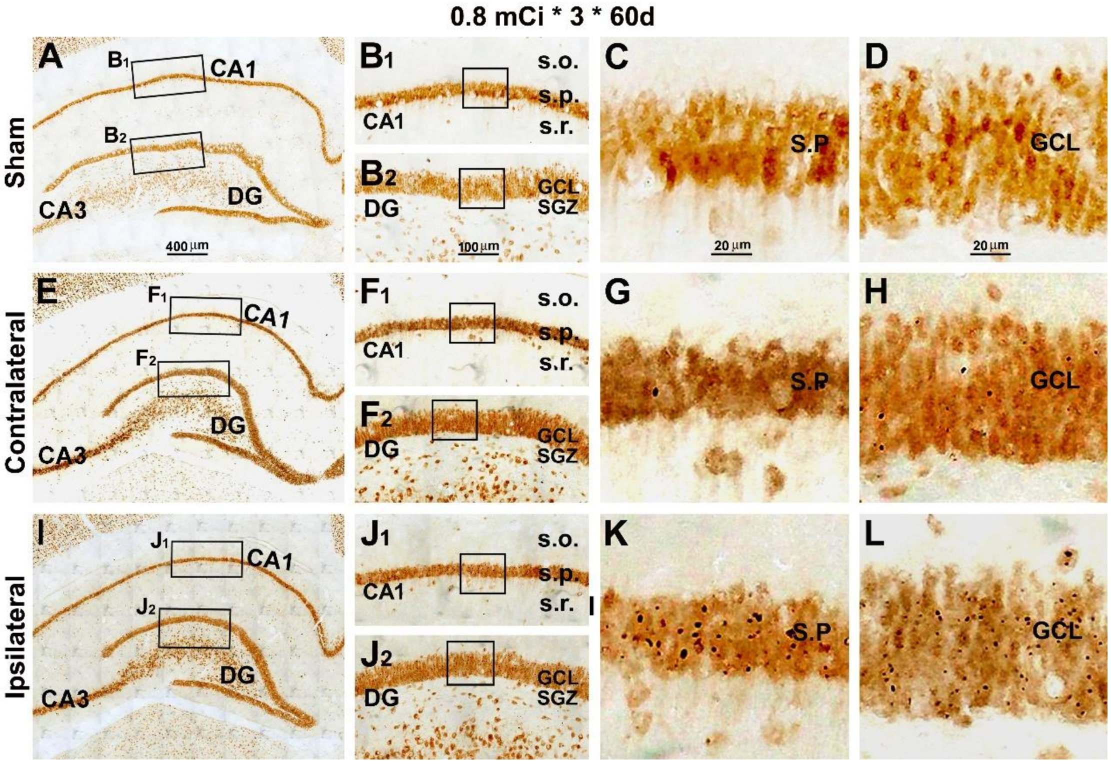

Supplemental Figure 2: NeuN immunolabeling in the hippocampus after 0.8 mCi radiation for 60 days relative to control. Panels (A-D) are images from sham control, and Panels (E-L) are from radiated animals. Note the occurrence of dark granule-like particles in the radiated hippocampus, which is more frequent in the ipsilateral side, as seen in the right panels at high magnification.

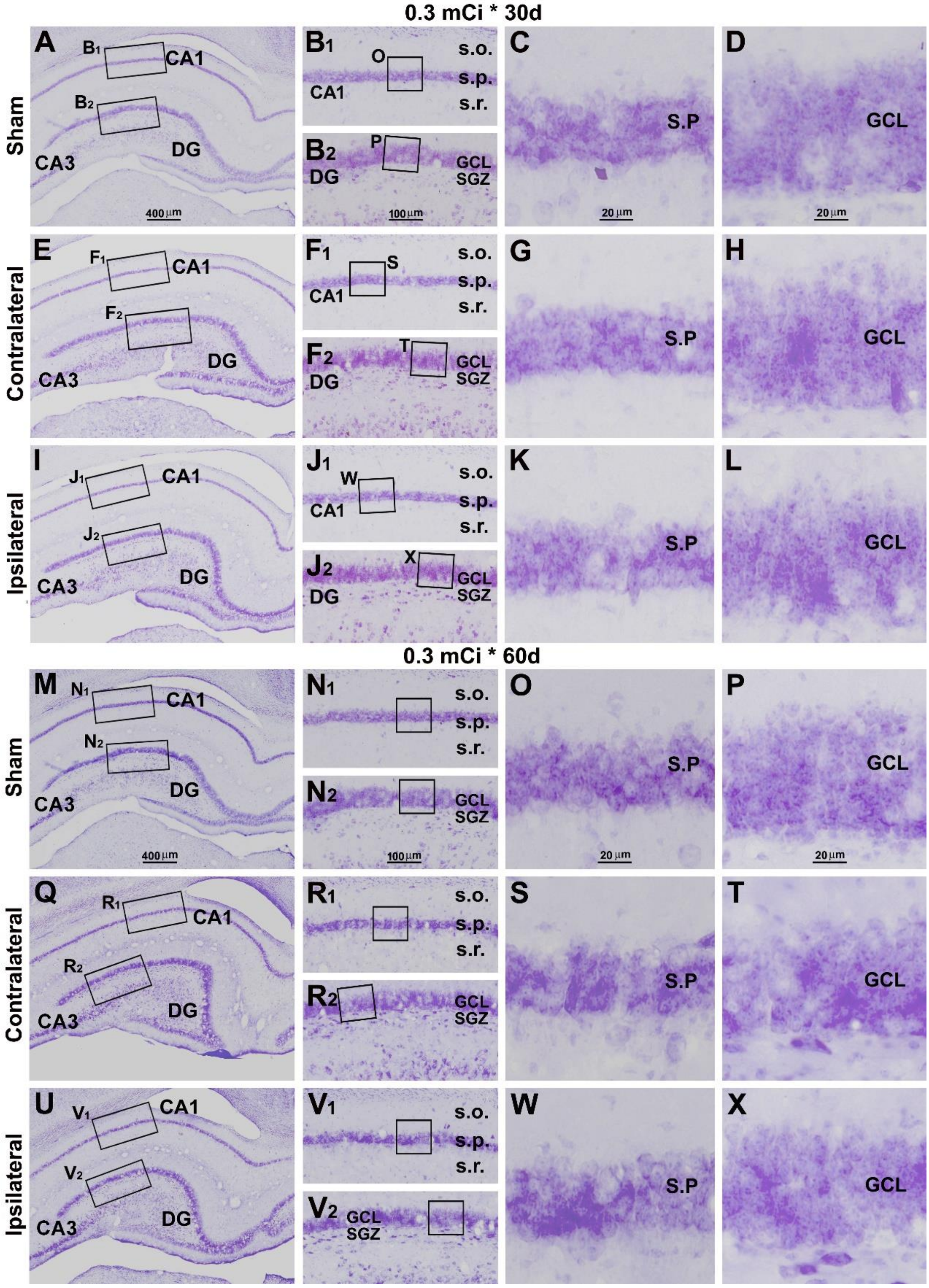

Supplemental Figure 3: Nissl stain of sections from animals with 0.3 mCi radiation for 30 days and 60 days relative to control.

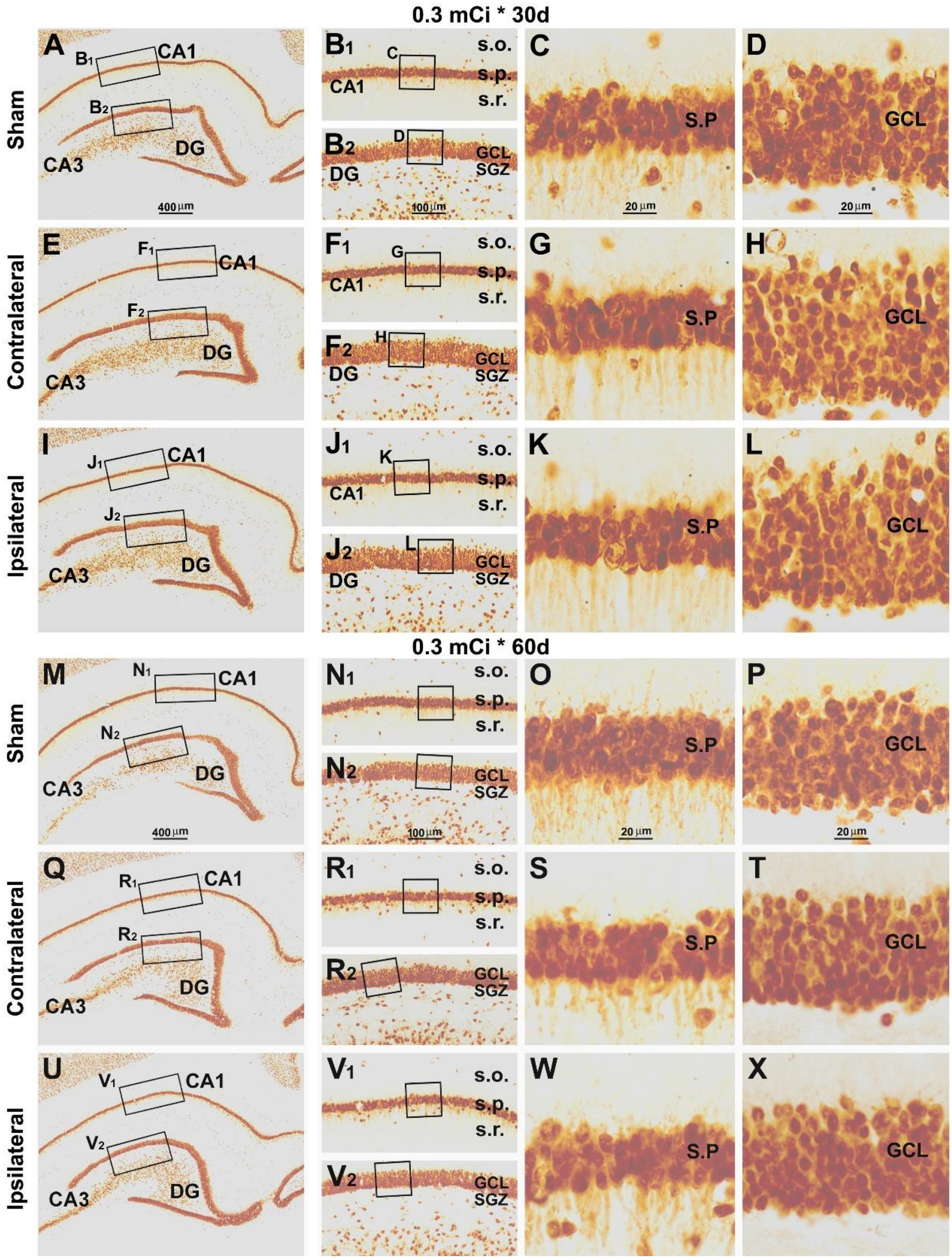

Supplemental Figure 4: Lack of effect on NeuN immunolabeling after 0.3 mCi radiation for 30 days and 60 days. The left panels are low-magnification images showing the orientation of Ammon's horn (CA1, CA3) and dentate gyrus (DG), with framed areas enlarged sequentially as indicated. No significant difference of NeuN+ can be detected in the GCL of DG and the s.p. of CA1 between the radiation and the sham groups. Scale bar = 400 μm in (A) applying to (E, I, M, Q, and U), equal to 100 μm for (B1, B2, F1, F2, J1, J2, N1, N2, R1, R2, V1, and V2), and 25 μm for (C, D, G, H, K, L, O, P, S, T, W, and X).

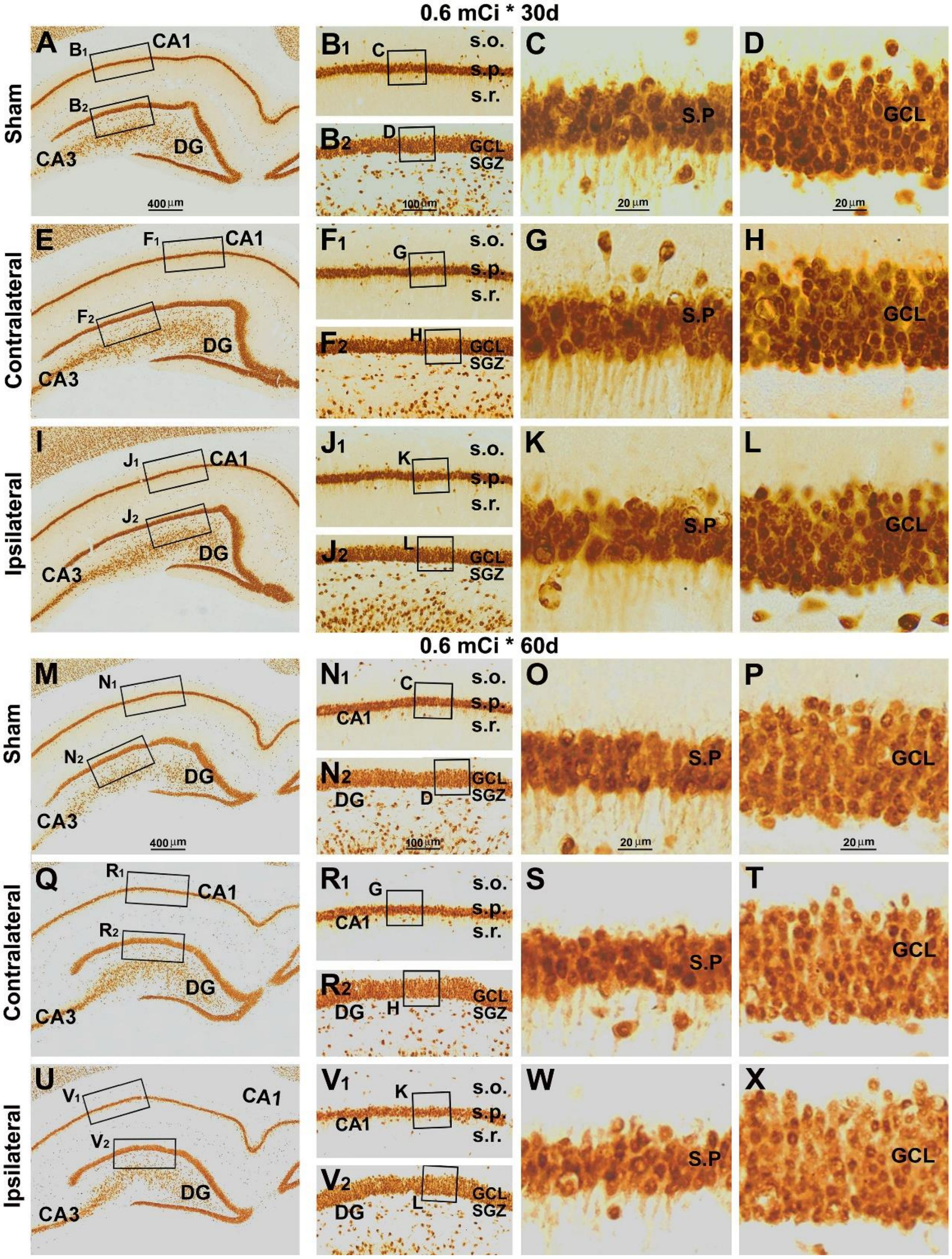

Supplemental Figure 5: Minimal effect on hippocampal NeuN immunolabeling after 0.6 mCi 125I radiation for 30 days and 60 days. Low-magnification (left panels) and enlarged areas are labeled accordingly (middle and right panels). No obvious difference in NeuN labeling could be detected between the two sides and the sham groups Scale bar = 400 µm in (A) applying to (E, I, M, Q, and U), equal to 100 µm for (B1, B2, F1, F2, J1, J2, N1, N2, R1, R2, V1, and V2), and 25 µm for (C, D, G, H, K, L, O, P, S, T, W, and X).

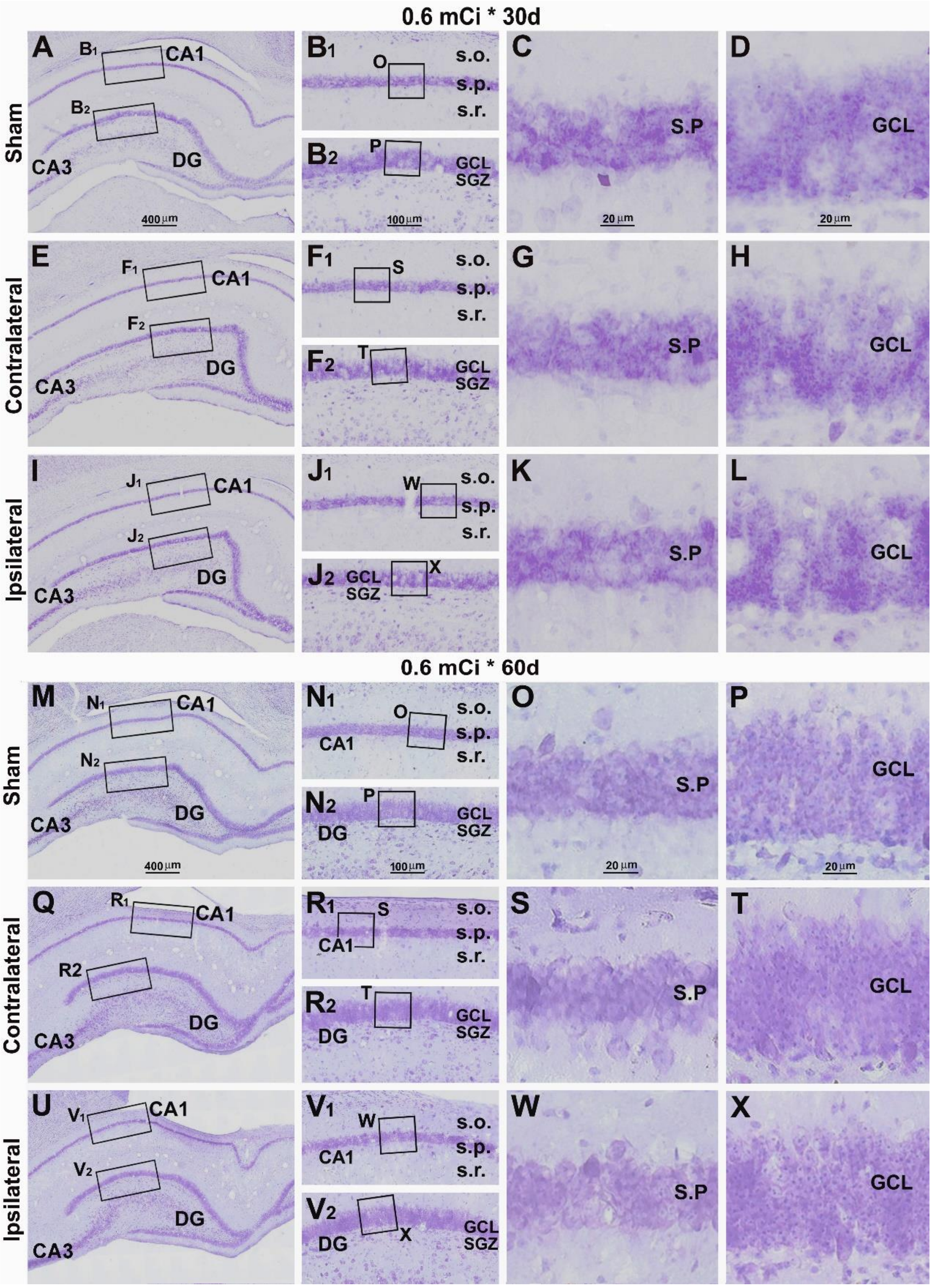

Supplemental Figure 6: Nissl stain of sections from animals with 0.6 mCi radiation for 30 days and 60 days relative to control.

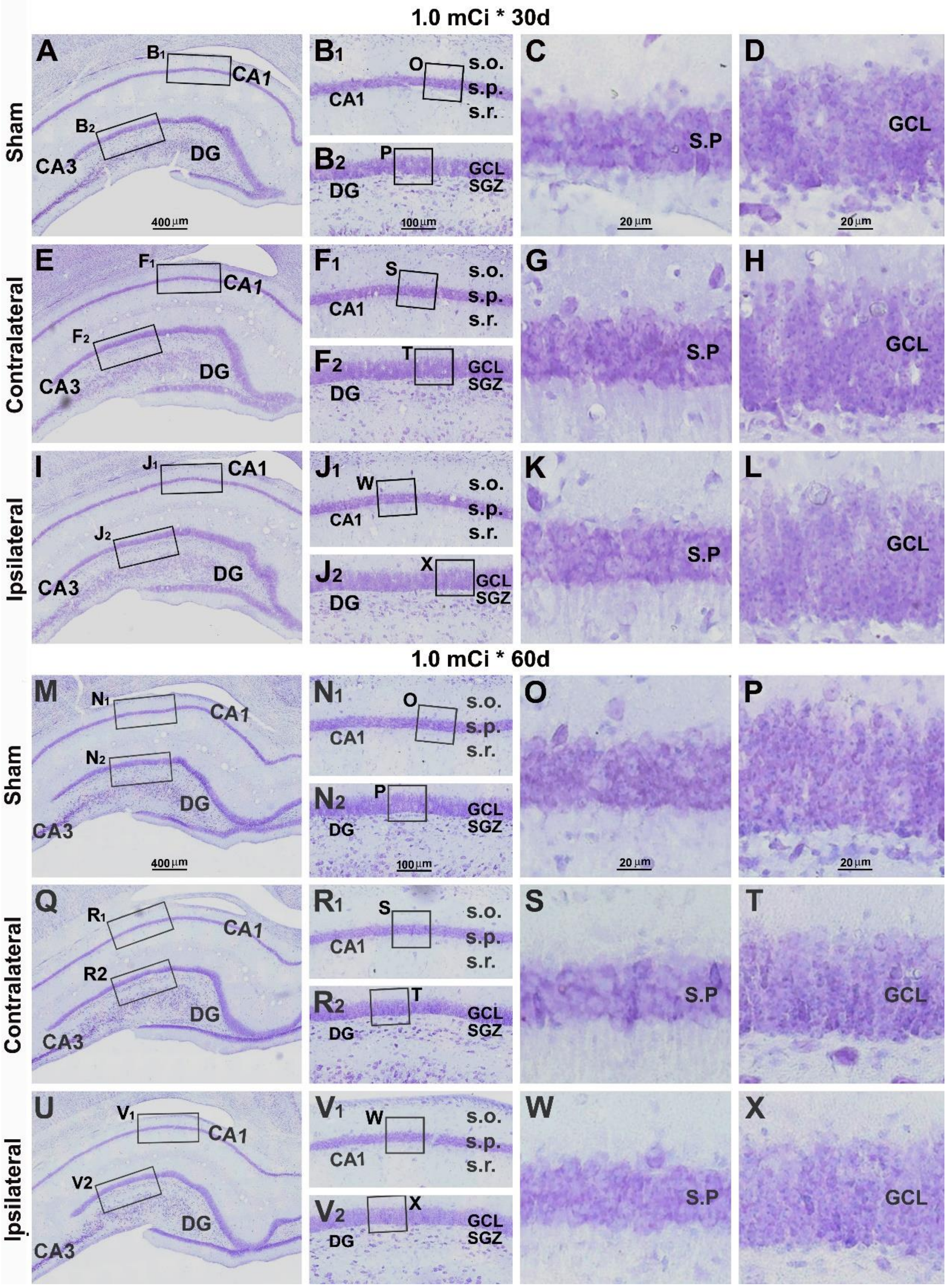

Supplemental Figure 7: Nissl stain of sections from animals with 1.0 mCi radiation for 30 days and 60 days relative to control.
